# In Vitro Reconstruction of Axonal Heat Sensing with a Photothermal Nerve‐on‐a‐Chip

**DOI:** 10.1002/smll.73577

**Published:** 2026-04-30

**Authors:** Koji Sakai, Yujiro Tanaka, Riku Takahashi, Toichiro Goto, Yosuke Mizuno, Taiki Otomo, Aya Tanaka

**Affiliations:** ^1^ Basic Research Laboratories NTT, Inc. Atsugi Kanagawa Japan; ^2^ NTT Bio‐Medical Informatics Research Center NTT, Inc. Atsugi Kanagawa Japan; ^3^ Device Technology Laboratories NTT, Inc. Atsugi Kanagawa Japan

**Keywords:** axon physiology, graphene, microelectrode array, microfluidic device, neuroengineering, organ‐on‐a‐chip, thermosensation

## Abstract

Axons are the initial sites of thermal signal detection and transduction, but their localized response to heat remains poorly characterized. Here, we present a photothermal nerve‐on‐a‐chip platform that reconstructs in vitro the key elements of axonal thermosensation. By integrating graphene‐based photothermal heaters with microelectrode arrays inside axon‐guiding microchannels, the platform enables localized, millisecond‐scale thermal stimulation and simultaneous extracellular recording of action potentials. Using this platform, we uncovered rapid and reproducible heat‐evoked responses in rat dorsal root ganglion neurons, including thermoreceptor‐mediated rapid desensitization. With its ability to distinguish stimulus‐locked and inter‐pulse activity, we also observed distinct thermal‐response profiles in human iPSC‐derived sensory neurons, characterized by delayed heat responsiveness. Our platform enables high‐resolution analysis of axonal temperature coding and is a scalable tool for exploring peripheral thermosensation in development and disease.

## Graphical abstract

A photothermal nerve‐on‐a‐chip platform is presented that enables precise, localized heating of sensory axons with simultaneous electrical recording. The system reveals millisecond‐scale dynamics of heat‐evoked neural activity and provides a new approach to study how temperature‐dependent ion channel behavior is translated into axonal firing.

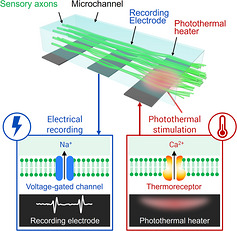

## 1 Introduction

By converting localized thermal stimuli into action potentials (APs) that rapidly reach the central nervous system, sensory axons initiate neural signaling in response to noxious heat. The ability to recognize such nociceptive signals is essential for protective reflexes and pain perception, and more broadly for thermal sensation and homeostatic adaptation [1, 2]. Understanding how these earliest steps of nociceptive signaling proceed at the axonal level is critical, because axons serve not only as conduits but also as sites of signal initiation and modulation. At the molecular level, heat‐sensitive ion channels such as transient receptor potential vanilloid 1 (TRPV1) open in response to noxious temperatures and trigger APs [3, 4]. To bridge the gap between molecular mechanisms and organismal responses, researchers have relied on cell culture models, including heterologous overexpression systems and primary rodent sensory neurons [5, 6, 7, 8, 9, 10, 11, 12, 13]. These preparations have been pivotal in dissecting how heat‐sensitive channels drive cellular firing responses, yet they are limited in their ability to capture human‐specific features. More recently, human‐induced pluripotent stem cell (iPSC)‐derived sensory neurons have emerged as an ethically favorable and physiologically relevant platform for studying nociception at the cellular level [14, 15, 16, 17, 18]. This advance provides a foundation for addressing unresolved questions on the axonal domain, particularly how human sensory axons encode noxious heat as part of thermal sensation and homeostatic adaptation.

Alongside the development of these cellular models, considerable progress has been made in methods for delivering thermal stimuli and recording neuronal activity. Patch‐clamp electrophysiology enables detailed analysis of ion channel and membrane responses [5, 6, 8, 9, 10, 11, 12], while microelectrode array (MEA) platforms support high‐throughput extracellular recordings [15, 16, 17, 18, 19, 20, 21]. In parallel, photothermal stimulation using nanomaterials, such as graphene [22], graphdiyne [23], MXene [24], silicon nanowires [25, 26, 27, 28], and gold nanorods [29, 30, 31, 32], has emerged as a precise and rapid approach for delivering localized heating with minimal electrical artifacts. These methods have been successfully used to evaluate thermal responsiveness in both central and peripheral neurons. However, direct characterization of sensory axonal responses to noxious heat has remained limited, because most studies have focused on somatic activity [22, 23, 24, 25, 26, 27, 28, 29, 30, 31, 32] or global bath heating [15, 16, 17, 18, 19, 20]. Addressing this gap requires experimental systems that can simultaneously deliver subcellular, millisecond‐scale thermal inputs and record axonal responses with high fidelity.

Here, we present a photothermal nerve‐on‐a‐chip (NoC) platform that integrates axon‐guiding microchannels, graphene‐based photothermal heaters, and MEA recordings. Microchannel‐based designs have been widely used to guide axonal growth in vitro [33, 34, 35, 36, 37], and they also confer an electrophysiological advantage by reducing electrical leakage and thereby amplifying extracellular potentials [38, 39, 40, 41, 42, 43]. This amplification permits reliable detection of small axonal spikes without the need for spike averaging. By leveraging this capability, our platform delivers localized, millisecond thermal stimulation at axons and simultaneously enables direct real‐time tracking of firing events. We used it to uncover rapid axonal responses to noxious heat in rat dorsal root ganglion neurons, including firing reductions consistent with thermoreceptor desensitization, and further identify distinct thermal‐response profiles in human iPSC‐derived sensory neurons. By reconstructing, in vitro, the sequence from heat detection to axonal propagation, our system constitutes a powerful tool for dissecting the spatiotemporal dynamics of thermal sensation at the cellular level.

## 2 Results

### 2.1 Fabrication of Photothermal Nerve‐on‐a‐Chip Device

**FIGURE 1:**
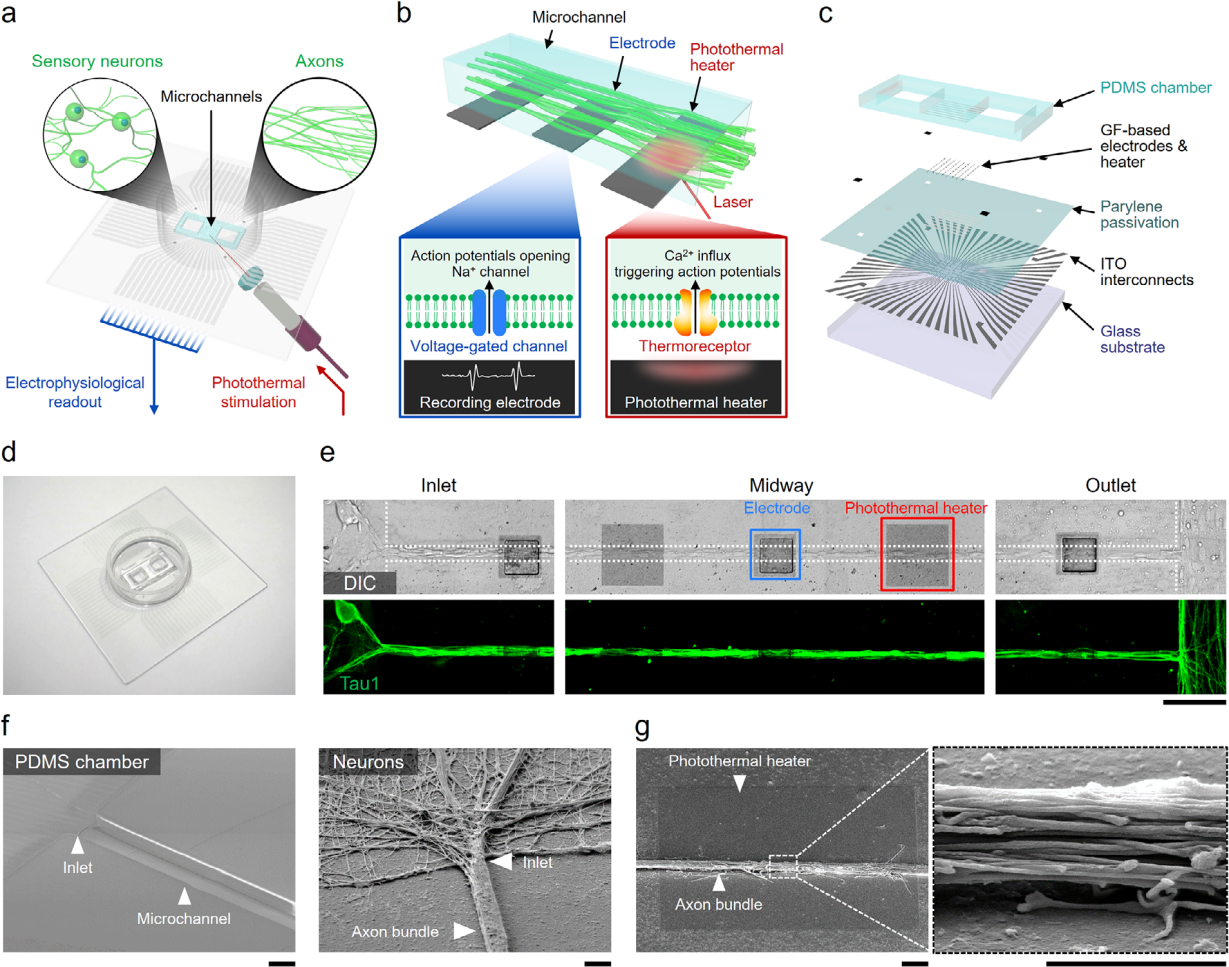
Design and fabrication of the photothermal nerve‐on‐a‐chip device. (a) Schematic of the experimental system integrating the laser‐irradiation and 64‐channel MEA for simultaneous stimulation and extracellular recording. (b) Enlarged view of a microchannel where axons pass over both the electrode and the photothermal heater, allowing detection of heat‐evoked action potentials from the same axon. (c) Composition of the NoC device combining a graphene flake (GF)‐based MEA with polydimethylsiloxane (PDMS) chamber. (d) Overview photograph of the fabricated NoC device. (e) Immunocytochemical and differential interference microscope (DIC) images of rat sensory neurons’ axons extending in a microchannel (20 µm width, 10 µm height, 3600 µm length). The white dotted lines indicate the wall of the PDMS chamber. (f) Scanning electron microscopy images showing bundled axons aligned along the entrance of the microchannel and then (g) extending linearly over the photothermal element. Scale bars,100 µm for **e**; 10 µm for (f, g).

The NoC device was fabricated by integrating a MEA substrate with axon‐guiding microchannels (Figure 1a–d; Figures S1–S6 and Tables S1–S4). Laser irradiation rapidly heats the heating element, and extracellular potentials are recorded concurrently from all 64 electrodes. Because the heater and electrodes share the same microchannel, APs generated in the same axon by a local temperature rise are captured reliably by the electrodes (Figure 1b). To keep the optical path clear for the laser, we selected highly transparent materials for every part of the MEA except the electrodes and heater. The MEA consisted of indium–tin‐oxide (ITO) feed lines coated with a parylene‐C insulating layer, with graphene flakes forming both the recording electrodes and photothermal heaters owing to their conductive and light‐absorbing properties (Figure 1c) [44]. The optical energy absorbed by the GF is converted into heat more efficiently than into electrical current [22]. This layer absorbs 17.5% of 785 nm light, which is enough for heating, yet is transparent enough for fluorescence imaging (Figure S6; Table S4). Although the GF layer decreases electrode impedance modestly (pristine ITO: 1.08 MΩ; GF‐coated: 0.65 MΩ at 1 kHz), the micro‐channels suppress leakage currents, enabling reliable AP recording. To capture an ensemble‐averaged response from axons within the axon bundle, the heater footprint was chosen to exceed the channel width.

The PDMS chamber isolates the growing axons from the cell soma. At a height below the soma diameter, somatic migration into the channel is prevented, and prior studies have shown that 10–50 µm‐wide channels reliably guide axons for millimeter‐scale outgrowth [36, 42, 43]. To resolve AP propagation as temporally separated waveforms, the channel was sized to accommodate eight electrodes at 450 µm intervals. Once seeded, neurons extend their axons from the soma compartment through these channels toward the distal outlet (Figure 1e). Scanning‐electron microscopy (SEM) at the channel entrance revealed that the axons fasciculate and align along the channel walls (Figure 1f; Figure S7). Inside the channel, the axon bundle runs straight across the electrodes and the photothermal heater while maintaining close contact with them (Figure 1g). Because the heater–electrode spacing is fixed, the AP propagation time between them can be converted directly into millisecond‐scale parameters such as conduction velocity and onset latency of AP generation.

### 2.2 Characterization of Photothermal Heater

**FIGURE 2:**
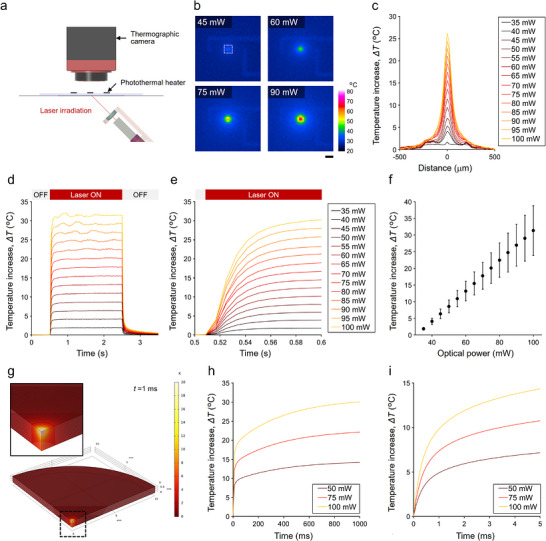
Thermal characterization of graphene‐based photothermal heater. (a) Schematic setup of measurement using a thermographic camera to evaluate temperature rise during laser irradiation. (b) Representative thermograms showing local heating upon laser exposure. The white dotted box indicates the heater. (c) Lateral temperature profiles with varied laser intensities, showing an increased temperature peak and a static dispersion. (d, e) Time course of temperature rise and saturation within ∼100 ms upon continuous laser stimulation. (f) Saturated temperature versus laser intensity, indicating a linear relationship. Note that a 45‐mW laser yielded a >6°C increase sufficient to activate TRPV1. (g) A representative finite element simulation result and (h) simulated time course of temperature increase, reproducing the rapid heating and saturation behavior of the photothermal heater. (i) Magnified temperature behavior, indicating a sufficient heating to exceed the TRPV1 threshold by millisecond pulses. Data are shown as mean ± SD (*n* = 7 heaters). Scale bars, 100 µm.

Prior to seeding neurons, the heating dynamics of the photothermal heater were quantified. As shown in Figure 2a, we illuminated the heater with a laser and then recorded its surface temperature in air to obtain a direct measurement of the heater response under minimal optical interference by using an infrared thermographic camera. The PDMS chamber was removed for this test. Representative thermograms (Figure 2b) show localized heating at the laser focus. Increasing the laser power raised the peak temperature (Figure 2c), whereas the lateral full width at half maximum (FWHM) of the thermal profile remained constant at ∼100 µm, independent of power (Figure S8). Comparable FWHMs were obtained with an unpatterned heater, indicating that heat dispersion is limited by the laser‐spot diameter rather than by heater geometry. Substituting the parylene‐C insulator with glass attenuated the temperature rise, confirming the thermal‐insulating capability of parylene‐C (Figure S8).

Next, we examined the transient temperature response. As depicted in Figure 2d,e, surface temperature rose within a few milliseconds and plateaued within ∼100 ms. The plateau temperature increased linearly with laser power (Figure 2f). To activate the TRPV1 channel, which is one of the main thermoreceptors in cultured sensory neurons, a ≥6°C increment above the 37°C culture baseline is required because the threshold of TRPV1 is 43°C. At 45 mW, the temperature rose by 6.4 ± 1.4°C in air and remained stable after 100 ms, providing an upper‐bound estimate of the thermal stimulus relative to the TRPV1 activation threshold. Finite‐element simulations reproduced the experimentally observed rapid rise and saturation (Figure 2g,h). Moreover, Figure 2i shows that even millisecond‐long laser pulses were sufficient to exceed the threshold. Although the 120‐Hz sampling rate of the infrared camera underestimated the intensities of the largest peaks, pulse‐width‐ and power‐dependent heating with millisecond pulses was confirmed experimentally (Figure S9). The temperature peak elicited by a 1‐ms pulse decayed to half‐maximum within 10–20 ms, irrespective of power. Together, these results demonstrate that millisecond laser pulses can deliver short‐time thermal stimuli sufficient to reach the activation range of TRPV1. The temperature rise measured in air likely overestimates the actual temperature at the axonal site in culture medium due to heat dissipation in the surrounding aqueous environment. Finite‐element simulations incorporating heat transfer conditions representative of water and PDMS (Figure S10) indicate that the temperature increase is reduced under these conditions, but remains within the range required to activate thermosensitive ion channels. While the simulation does not explicitly incorporate the detailed microchannel geometry, it captures the effect of heat dissipation in the surrounding medium and provides a conservative estimate of the thermal conditions.

### 2.3 Axonal Responses to Continuous Thermal Stimulation

**FIGURE 3:**
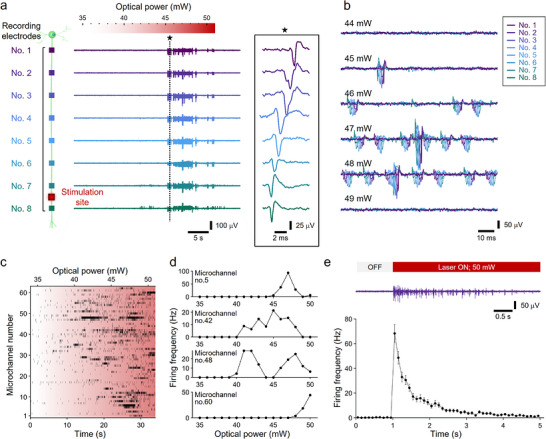
Diverse axonal responses to continuous photothermal stimulation. (a) Representative signals recorded from eight electrodes aligned in the same microchannel upon continuous laser irradiation with a gradual power increment. The magnified view (★) shows a single evoked event orderly propagating across the electrodes. (b) Spatiotemporal mapping of evoked spikes, showing both retrograde and anterograde AP propagations along axons. (c) Raster plots from active microchannels showing raising temperature evoked neural firing with variability in activation thresholds and firing onset. (d) Examples of distinct firing frequency trends per microchannel. (e) A representative signal and an averaged trend of firing frequency upon continuous stimulation with a static laser power at 50 mW, indicating a rapid decline in firing frequency within a few milliseconds after stimulation. Data are shown as mean ± SE (*n* = 38 microchannels).

We then quantified the axonal responses to localized heating. In preliminary whole‐bath experiments, we verified that rat dorsal root ganglion (DRG) cultures increase their firing sharply above 43°C—the threshold for TRPV1 activation (Figure S11). Since noxious heat is detected at the distal axon in vivo and then APs propagate antidromically toward the soma, we irradiated the heater farthest from the cell bodies to replicate this geometry (Figure 3a). Thermographic measurements showed that a 45‐mW laser elevates local temperature by 6°C in air, exceeding the TRPV1 threshold. Therefore, the laser power was ramped up from 35 mW, increasing by 1 mW every 2 s. To avoid thermal injury, all recordings were performed within a non‐injurious window (≤ 60 mW; ΔT ≤ 13°C in air; exposure 2 s). This window was established from 24‐h viability assays and is detailed in Figure S12. Representative recorded signals are presented in Figure 3a. Below 45 mW, only sporadic spontaneous spikes were detected, whereas firing frequency dramatically rose when the power exceeded 45 mW. As indicated in the inset of Figure 3a, each event displayed the bipolar or triphasic negative peaks characteristic of extracellularly recorded APs. The orderly time lags between electrodes confirmed that the APs propagated along the axons. As illustrated in Figure 3b, most APs traveled antidromically from the stimulus site, although the rebound of AP propagation at the soma occasionally generated orthodromic AP propagations [45, 46]. When the stimulus site was the midpoint of the microchannel, APs propagated in both directions (Figure S13). Note that the electrodes recorded APs simultaneously from multiple axons. Since many axons fasciculated within a single microchannel, one stimulus activated multiple axons, producing a mixture of AP waveforms (Figure 3b). Nonetheless, the signal amplification within the microchannel allowed us to track individual AP propagations.

A remarkable feature of the thermal responses was that the power threshold for excitation varied from one microchannel to another. Figure 3c presents raster plots from all responsive channels as laser power was incrementally increased. In most channels, firing increased above 40 mW, whereas the threshold ranged broadly from 35 mW to 50 mW (Figure S14). Representative trends of the response (Figure 3d) show that both the threshold and the duration of the elevated firing differed among microchannels, reflecting axon‐to‐axon variability in the amount and composition of thermoreceptors. Because of this dispersion, subsequent quantitative results are presented as mean ± SE and focus on trends in the population average.

Despite the variability among microchannels, a highly consistent feature was that the firing rate decayed rapidly. To quantify that decline, we applied a continuous thermal stimulus at a fixed power and recorded the response. As shown in Figure 3e, firing fell steeply within several seconds. TRPV1 channels are known to undergo rapid, Ca^2^^+^‐dependent desensitization that lowers their activation threshold on a millisecond‐to‐second time scale, consistent with the observed decay [8, 9, 10, 11]. These results demonstrate that the present platform can reliably capture the loss of excitability associated with thermoreceptor desensitization. Importantly, since the decline begins within sub‐second timescales, the recording system must have millisecond resolution to track these fast changes accurately.

### 2.4 Millisecond Thermal Pulses Reveal Rapid Axonal Excitability

**FIGURE 4:**
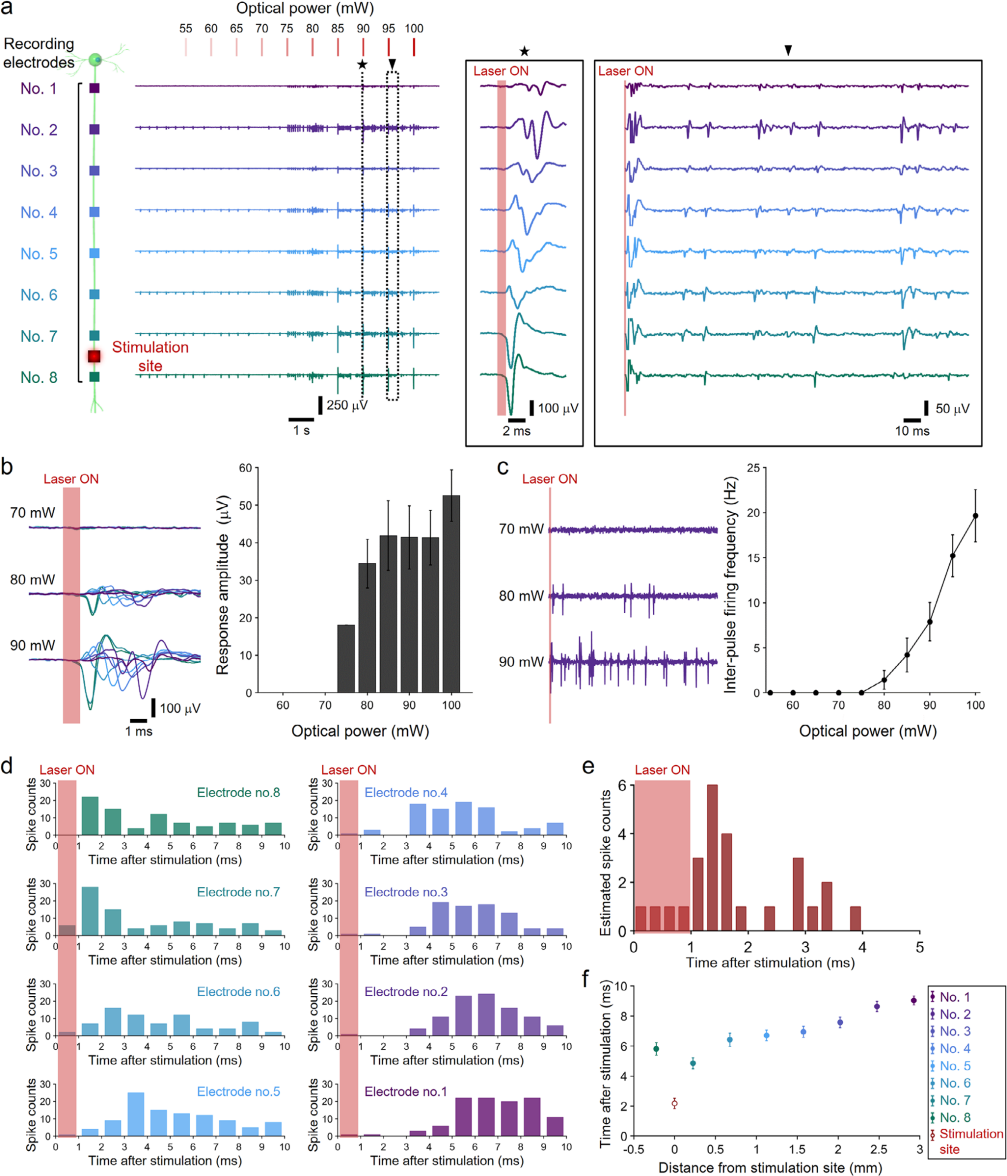
Millisecond photothermal pulses elicit fast and precise axonal responses. (a) Representative traces with 1 ms laser pulses of increasing intensity. Magnified waveforms show evoked action potentials immediately after pulse stimulation (★) and inter‐pulse firing (▼). (b) Amplitude of pulse‐locked response increased with intensity, indicating recruitment of more axons. (c) Inter‐pulse firing frequency also increased with laser intensity. (d) Histogram of spike timing in pulse‐locked response across electrodes shows antidromic action potential propagations along axons. (e) Estimated histogram of action potential onset latency from conduction time between electrodes. (f) Mean spike timing plotted against electrode position shows the accumulation of temporal jitter with distance. Data are shown as mean ± SE (*n* = 18 microchannels for (b, c); *n* > 30 stimuli for f).

To probe axonal responsiveness on sub‐second timescales, we delivered photothermal 1‐ms‐wide pulses while incrementally increasing laser power. To avoid thermal injury, all recordings were performed within a non‐injurious window (≤ 100 mW; ΔT ≤ 10°C in air; exposure 1 ms). Representative recorded signals are shown in Figure 4a. Beyond a certain laser power, APs were triggered immediately after each pulse. The APs propagated between electrodes with time lags, as was observed under continuous heating. Notably, the pulse‐evoked APs originated at the stimulus site and therefore appeared antidromic on the electrode array, whereas during continuous heating, both antidromic and orthodromic propagations were observed (antidromic 83 ± 3%). When the heater closest to the soma was stimulated, propagation was orthodromic (Figure S13). Thus, millisecond pulses selectively activate the physiologically relevant AP propagations originating from the distal part of the axon. Although finite‐element simulations indicated that the elevated temperature by a 1‐ms pulse decayed within 10–20 ms, the firing rate remained higher than baseline throughout the 1‐s inter‐pulse interval. Given the rapid desensitization of thermoreceptors, it is unlikely that the residual sub‐threshold heat reopened thermoreceptors. In contrast, intracellular Ca^2^^+^ has been reported to remain above baseline for several seconds after a 10‐ms thermal pulse [8, 13], suggesting that the remaining intracellular Ca^2^^+^ may promote firing between stimuli. Therefore, in the subsequent analysis, we distinguished the pulse‐locked response from the inter‐pulse activity to examine these two phases of excitability.

Both pulse‐locked and inter‐pulse activity increased with laser power (Figure 4b,c). Since each microchannel contained multiple axons, the pulse‐locked response is a compound waveform of spikes from several axons. Its peak amplitude, therefore, reflects the number of axons that activated simultaneously. Consistent with this interpretation, the peak amplitude rose as power increased (Figure 4b), and the fraction of microchannels exhibiting the response, which is called the response probability, showed a similar trend (Figure S15). In contrast, increasing the laser power had minimal effect on AP conduction velocity (Figure S15). The measured velocity was 0.68 m s^−^^1^, which corresponds to typical values for cultured rat DRG neurons [21, 42], indicating AP conduction velocity is determined by membrane properties and is independent of stimulus intensity. Inter‐pulse firing also increased with power (Figure 4c). The parallel increment of pulse‐locked and inter‐pulse activity suggests that activated axons remained in an excitable state between stimuli, possibly owing to residual intracellular Ca^2^^+^. The excitation threshold of both pulse‐locked and inter‐pulse activity was between 75 and 80 mW. Finite‐element analysis predicted that a 1‐ms pulse at 75 mW would raise the temperature by 7.4°C in air (Figure 2i), which surpasses the 43°C activation threshold of TRPV1, and this supports the conclusion that the experimentally observed threshold is consistent with TRPV1 gating kinetics.

To examine the temporal sequence from heating to AP initiation, we compared the latencies of pulse‐locked activity recorded on each electrode (Figure 4d–f). The latency histograms (Figure 4d) reveal a progressive delay and a concomitant broadening of the distribution, as the recording site was positioned farther from the heater. This broadening was probably because small differences in conduction velocity among axons accumulate with distance. Consequently, compound waveforms recorded near the stimulus site were sharp, whereas those recorded distally exhibited multiphasic shapes (inset, Figure 4a). The measured latency reflects two components: (i) the conduction delay along the axon and (ii) the onset latency of AP initiation. To isolate the latter, we back‐extrapolated the conduction time between the electrode and heater (Figure S16). Using the conduction time between electrodes nos. 3 and 6, we constructed a histogram of the estimated AP initiation times (Figure 4e). The distribution is clustered just after the end of the laser pulse, peaking 0.25–0.5 ms later. This interval agrees with the reported values for the delay between a rapid temperature rise and TRPV1 channel opening [5, 6, 7], suggesting that the platform can resolve the channel‐opening interval from the heating. Since the compound waveform was analyzed, the overlap of APs in individual axons may introduce an error in the estimation. Indeed, a plot of mean latency versus electrode position deviates from complete linearity (Figure 4f), implying distortion of the compound waveform by overlapping AP waveforms. Using narrower micro‐channels guiding fewer axons should improve accuracy [41, 47]. Nevertheless, the present results demonstrate that changes in onset latency can be estimated with millisecond precision.

### 2.5 Desensitization Dynamics Under Repetitive Millisecond Thermal Stimulation

**FIGURE 5:**
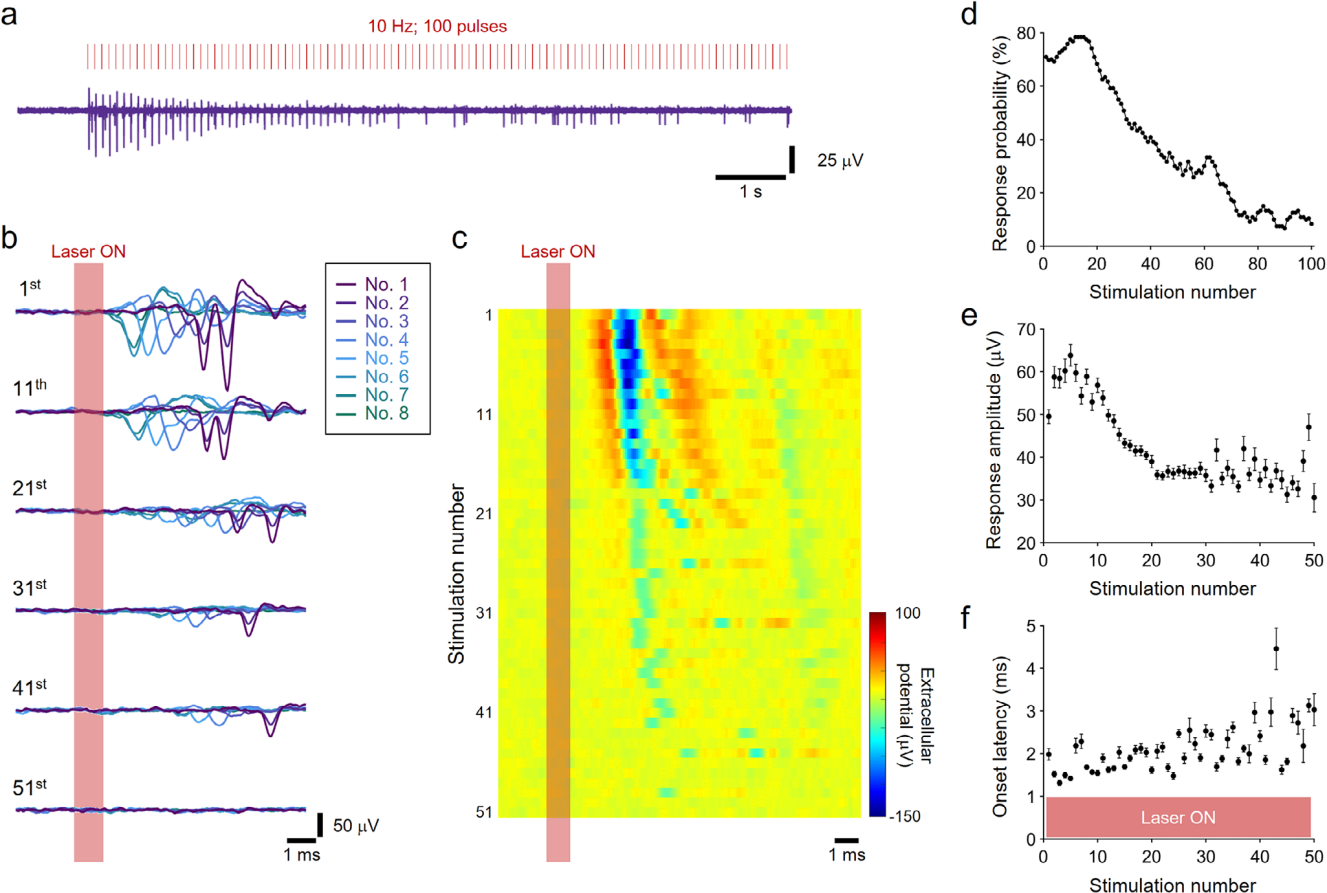
Progressive decline in excitability under repetitive photothermal pulses. (a) A representative trace of axonal responses to 10 Hz laser pulses (1 ms, 100 mW). (b, c) Representative traces and an extracellular potential colormap of pulse‐locked responses to each stimulus, showing a decrease in response amplitude and latency shift. (d) Response probability increased initially and then declined with further repetitions. (e) Amplitude peaked at the fifth pulse and dropped significantly by the 20th pulse. (f) Onset latency gradually increased with repeated stimulation, reflecting TRPV1 desensitization. Note that these changes occurred without alteration in conduction velocity, indicating preserved axonal transmission. Data are shown as mean ± SE (*n* = 24 microchannels for (d); *n* > 3 microchannels for each stimulation number in (e, f).

By leveraging the millisecond resolution of our platform, we quantified how TRPV1‐mediated desensitization attenuates axonal responses during repetitive stimulation. Here, the heater most distal to the soma was driven with 1 ms laser pulses at 10 Hz and 100 mW—an intensity that reliably elicited APs. Representative trace and pulse‐locked responses are shown in Figure 5a–c. As the stimulation repeated, the amplitude diminished, and the response latency was prolonged. The changes were evaluated with three metrics: response probability, peak amplitude, and onset latency (Figure 5d–f). Response probability rose during the first 10^th^ pulses, then declined progressively with further stimulation (Figure 5d). Peak amplitude rose until the fifth pulse, then dropped sharply until the 20th pulse and thereafter remained at the lower amplitude limit (Figure 5e). The early rise over the first ‐5^th^ pulses likely reflects intracellular Ca^2^^+^ accumulation or a transient lowering of the activation threshold, whereas the subsequent decline is consistent with Ca^2^^+^‐dependent TRPV1 desensitization. When onset latency was back extrapolated, it was found to have increased throughout the stimulus train (Figure 5f), while the conduction velocity remained unchanged (Figure S14). This invariance of the conduction velocity is physiologically plausible because AP propagation depends on voltage‐gated ion channels rather than TRPV1 [48]. Therefore, the progressive response delay in repeated stimulation originates from the prolonged onset latency, not from slower propagation. This latency increase is attributable to a reduced number of available TRPV1 channels and a slower channel‐opening rate. These findings demonstrate that the device resolves the entire sequence from TRPV1 opening to AP initiation and propagation, enabling real‐time tracking of desensitization‐mediated changes in axonal excitability.

### 2.6 Thermal Responsiveness of Human iPSC‐Derived Sensory Neurons

**FIGURE 6:**
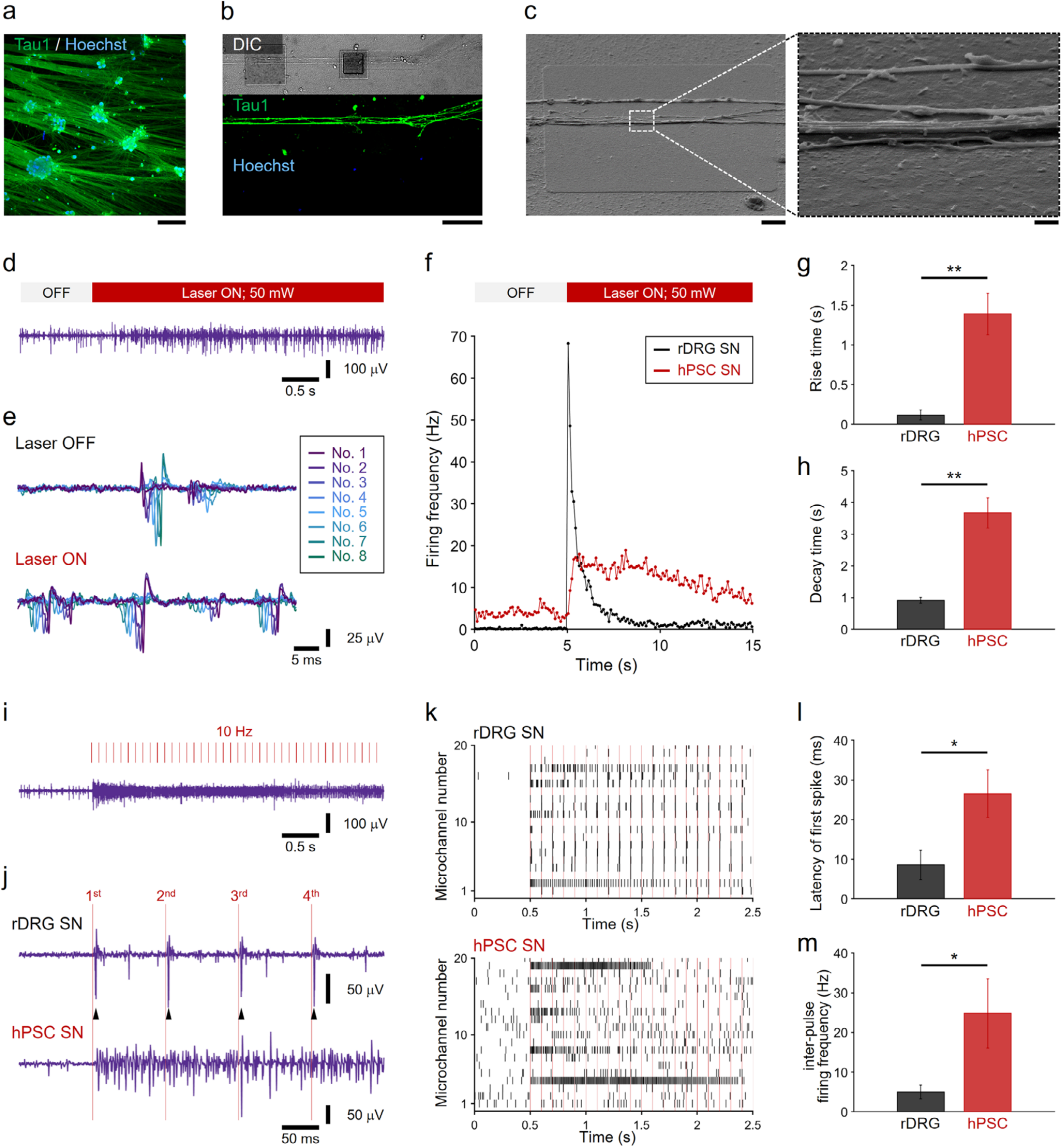
Comparison of thermal responsiveness between rat DRG neurons and human iPSC‐derived sensory neurons. (a) Immunocytochemical image of human iPSC‐derived sensory neurons (hPSC SN) in the soma compartment. (b) Immunocytochemical and differential interference microscope (DIC) images of extending axons at the farthest heater from somata. (c) Scanning electron microscopy image at the same heater in b. These images were captured after 10 Hz stimulation, indicating that the axons remain structurally intact after stimulation. (d, e) Representative traces upon continuous stimulation showing firing evoked by thermal stimulations. (f) The time course of firing frequency in response to the continuous stimulation, indicating a slower and weaker firing increase in hPSC SN than rDRG SN. (g, h) hPSC SNs exhibited longer rise time and slower decay in the trend of firing frequency. (i, j) Representative traces upon repeated 1 ms pulse stimulations. (k) Raster plot of spike times showing the high fidelity of the pulse‐locked response in rDRG SN, in contrast to the higher inter‐pulse firing in hPSC SN. (l, m) Quantification of these features showing the lower amplitudes in the initial response and higher inter‐pulse firing frequency in hPSC SN. These results suggest immature thermal coding due to developmental differences in ion channel expression. Data are shown as mean ± SE. *n* = 22 (rDRG) and 38 (hPSC) microchannels for (f–h); *n* = 18 (rDRG) and 11 (hPSC) microchannels for (l, m). Scale bars, 100 µm for (a, b); 10 µm for (c). ^*^ *p* < 0.05 and ^**^ *p* < 0.01; Welch's *t*‐test.

To extend the platform to investigations more relevant to physiology and disease, we assessed thermal excitability in human iPSC‐derived sensory neurons (hPSC‐SNs). Sensory neuron progenitors were seeded into the soma compartment and maintained for 4 weeks. These neurons have been reported to express TRPV1 and to respond to temperature elevations around 43°C [15]. As shown in Figure 6a, the cultures contained few Schwann cells, yielding relatively pure neuronal cultures, unlike rat primary DRG cultures (rDRG‐SNs). Although axonal outgrowth was slower than in rDRG‐SNs (Figure 6b), the neuron‐enriched culture allowed for direct observation of axonal morphology. The SEM image at a heater shows that axons remained intact after repeated thermal stimulation (Figure 6c). All recordings were performed on 4‐week cultures in which axons reached the microchannel outlet.

The excitability profile of hPSC‐SNs differed markedly from that of rDRG‐SNs. Before stimulation, rDRG‐SNs were almost silent, whereas hPSC‐SNs exhibited distinct spontaneous firing (0.2 Hz in rDRG‐SNs vs. 5.8 Hz in hPSC‐SNs; Figure S17). Spontaneous firing has been reported in hPSC‐SNs [16, 17, 18], while expression of ion channels appears lower in hPSC‐SNs than in mature DRG neurons [14, 16]. Continuous laser irradiation increased firing in hPSC‐SNs (Figure 6e), whereas their peak rate (18.0 Hz) was lower than that of rDRG‐SNs (68.3 Hz). The rise time to peak firing was markedly longer (Figure 6g). Moreover, the decay of firing was also slower (Figure 6f,h), suggesting delayed desensitization. Notably, hPSC‐SNs have been reported to express lower levels of TRPV1 mRNA compared with native human DRG neurons, suggesting the immaturity of hPSC‐SNs [14, 16]. Therefore, these features suggest lower density or reduced sensitivity of thermoreceptors in hPSC‐SNs.

Differences in thermal responsiveness were further highlighted under pulsed stimulation (Figure 6i–m). Although hPSC‐SNs responded to 1‐ms pulses at 10 Hz, they didn't exhibit pulse‐locked responses, unlike rDRG‐SNs (Figure 6j). Raster plots (Figure 6k) show that rDRG‐SNs reliably tracked each stimulus pulse, whereas hPSC‐SNs exhibited prominent inter‐pulse firing, which obscured the identification of the pulse‐locked events. First‐spike latencies were longer, whereas spike amplitudes were comparable in hPSC‐SNs (Figure 6l; Figure S17). The absence of a pulse‐locked response suggests that firing was poorly synchronized. Despite the weak pulse‐locked responses, inter‐pulse firing was significantly higher in hPSC‐SNs (24.8 Hz) than in rDRG‐SNs (5.0 Hz; Figure 6m). This elevated activity decayed over seconds, implying that thermoreceptor desensitization proceeded (Figure S17). Thus, the inter‐pulse firing likely originates from sustained depolarization rather than persistent thermoreceptor opening, suggesting immature Ca^2^^+^ handling and repolarization mechanisms that could delay the clearance of intracellular Ca^2^^+^. Taken together, compared with rDRG‐SNs, hPSC‐SNs exhibit higher spontaneous firing, slower and weaker heat‐evoked responses, and prolonged inter‐pulse excitability, likely reflecting developmental immaturity and lower or less sensitive thermoreceptor expression.

## 3 Discussion

The core strength of this NoC platform is its axon‐focused readout with millisecond precision. By confining the photothermal hotspot and the electrodes within the same microchannel, recordings are predominantly axonal and largely free from somatic contamination. In this configuration, we stimulated a defined intra‐axonal segment and captured the sequence from local heat transduction to action‐potential initiation and propagation, enabling quantitative measurements such as onset latency and conduction velocity with high reproducibility. Although patch‐clamp electrophysiology provides high temporal resolution for evaluating spike fidelity, direct axonal recordings are technically challenging due to the small axonal diameter [49]. Bulk‐heating MEA approaches predominantly report somatic spiking and provide poor localization of the initiation site [15, 19, 20]. Compared with soma‐centric patch clamp or bulk‐heating planar MEAs, our geometry restricts both the stimulus and the recording to a defined axonal compartment. This configuration improves the signal‐to‐noise ratio, enabling the device to resolve initiation and conduction with high fidelity and to track heat‐evoked axonal events over time and across adjacent electrodes.

With this temporal fidelity, two phenomena emerged robustly: (i) a second‐scale decline in heat‐evoked firing consistent with rapid desensitization of thermoreceptors, and (ii) millisecond‐scale tracking of brief inputs, including sharp, stimulus‐locked responses to 1‐ms pulses. rDRG‐SNs responded sharply to 1‐ms pulses and remained silent otherwise, indicating their ability to encode sub‐millisecond thermal information, whereas hPSC‐SNs showed weaker contrast, consistent with immaturity in their temperature‐processing properties. The system further allowed estimation of sub‐millisecond onset latencies following rapid heating, dissociating channel opening and conduction delays, and revealed stable conduction velocities even as desensitization progressed under repetitive stimulation. Together, these results show that the device can resolve rapid gain changes in axonal excitability and encode transient thermal information with high fidelity—capabilities that are valuable for both pharmacological assays and computational analyses of thermal coding. Although these dynamics are consistent with thermoreceptor‐mediated responses, additional temperature‐sensitive mechanisms, including TREK channels, inward‐rectifier potassium channels, and the intrinsic temperature dependence of voltage‐gated Na^+^ and K^+^ channels, may also contribute to the observed axonal excitability[1]. More broadly, the present platform provides a means to probe how temperature‐dependent changes in ion channel gating are translated into axonal firing dynamics with millisecond resolution.

Using hPSC‐SNs offers distinct advantages. TRPV1 is the principal detector of noxious heat and a key node in pain processing [1, 2, 3, 4]. Prior studies report lower TRPV1 expression in hPSC‐SNs than in native human DRG and co‐expression of multiple thermoreceptors (e.g., TRPM8, TRPA1), consistent with an immature, heterogeneous thermal phenotype [15, 16]. Such heterogeneity has been proposed to support population‐level encoding of temperature and pain and varies with in‐vitro maturation stage, aligning with our observation of weaker, slower heat‐evoked responses in hSNs [16, 50]. This immaturity is therefore useful rather than limiting; it enables modeling of developmental trajectories and desensitization‐driven adaptation in a human context. Moreover, MEA‐based assays using hPSC‐SNs have already been applied to analgesic screening across pain‐related thermoreceptor pathways, underscoring the translational value of a minimally invasive, high‐throughput platform [15]. Taken together with our axon‐focused readouts, these features position hPSC‐SNs as a practical human model for mechanistic studies of thermal coding and for scalable pharmacological screening.

The most distinctive feature of this NoC device lies in its ability to reconstruct, in vitro, the sequence from thermal nociception to axonal action‐potential initiation and propagation with millisecond precision. Notably, the platform enables a robust evaluation of heat‐evoked axonal responsiveness without material, wavelength, or irradiation optimization, underscoring its practicality and reproducibility. Meanwhile, the fabrication route based on spin‐coating photothermal nanomaterials remains broadly extensible. Alternative absorbers (e.g., MXene, gold nanorods, silicon nanowires) [22, 23, 24, 25, 26, 27, 28, 29, 30, 31] and different optical bands can tailor heating efficiency and depth. Microchannel width and channel count can be tuned to guide fewer axons for single‐axon resolution or scaled up for high‐throughput screening. These components are compatible with emerging nerve‐on‐a‐chip formats, including 3D cultures and organoid‐based sensory models [51, 52, 53, 54], whose primary advantage is their tissue‐like architecture and spatial organization. Integrating our axon‐focused perturbations with such formats situates localized stimulation in a more anatomically realistic context. Taken together, the simple integration of photothermal nanomaterials, axon‐guiding microchannels, and MEA recording provides a versatile platform to quantify rapid thermal responses in axons; we believe it will deepen our understanding of peripheral thermosensation and support scalable pharmacological and computational studies.

## 4 Experimental Section

### 4.1 Fabrication and Assembly of the Nerve‐on‐a‐Chip Device

The MEA substrate and PDMS chamber were fabricated using standard photolithography, following procedures as previously reported [42, 55]. Schematic diagrams of the fabrication steps are shown in Figures S2–S5, and the structural parameters are summarized in Tables S1–S3.

The MEA was fabricated as follows. A glass substrate coated with ITO (<15 ohm/sq) was patterned with feedlines using a positive photoresist (S‐1813; Rohm and Haas). UV exposure was performed through a chromium photomask using a UV exposure (UV Kub, Beams), followed by development with Microposit 351 Developer (Rohm and Haas). Unprotected ITO regions were etched using an ITO etchant (ITO‐02; Kanto Chemical), leaving the patterned feedlines. The photoresist was removed with acetone and ethanol (Kanto Chemical), after which a 1.5 µm‐thick parylene‐C layer was deposited via chemical vapor deposition (CVD; SCS, Labcoater PSD2010). Next, openings for electrodes and contact pads were patterned using S‐1813 photoresist. A mask aligner (ES410; Nanometric Technology) was used to align these patterns with the feedlines. The parylene‐C layer was etched by reactive ion etching (RIE) with oxygen plasma, using the photoresist as a mask. After removing the photoresist with acetone and ethanol, a graphene ink (793663; Sigma‐Aldrich) was spin‐coated at 4000 rpm. This commercial ink contains 2.4 wt% GFs dispersed in a cyclohexane/terpineol solvent, with ethyl cellulose added as a binder. To prevent any flakes from leaching into the culture medium, the substrate was rinsed with acetone and ethanol, leaving a 30 nm‐thick GF layer (Figure S6). A second photolithography step using S‐1813 defined the electrode and heater patterns, and oxygen plasma RIE was used to remove excess GFs. After final rinsing, a 20 mm‐diameter glass ring was bonded to the MEA using PDMS (Silpot 184; Dow Corning Toray) to form a reservoir for cell culture medium. Prior to assembling the PDMS chamber, the MEA was coated with 0.1% polyethyleneimine (Sigma‐Aldrich) to enhance cell adhesion.

The PDMS chamber with microchannels was fabricated by soft lithography using an SU‐8 mold with two‐layer structures of different heights. A silicon substrate was first rendered hydrophilic with an oxygen plasma treatment, followed by spin‐coating with hexamethyldisilazane (HMDS; Tokyo Chemical Industry) to promote SU‐8 adhesion. After HMDS evaporation, SU‐8 3010 (Microchem) was spin‐coated at 4000 rpm and exposed using a maskless writer (PALETTE; Neoark) to define the microchannel pattern. Development was performed using SU‐8 Developer (Microchem). A second layer of SU‐8 3050 was then spin‐coated at 1000 rpm to form the soma compartment. The height of this compartment was designed to exceed the cell body diameter to promote neuron seeding near the channel inlets. The finished SU‐8 mold was coated with parylene‐C to facilitate PDMS release. PDMS prepolymer (10:1 base:curing agent) was poured onto the mold, and cured at 80 °C for 2 h. The cured PDMS was peeled off, and the soma compartment was punched out manually.

The integration of the PDMS chamber onto the MEA was performed as follows. To suppress cell adhesion to the PDMS surface, the chamber was coated with 1% bovine serum albumin (BSA; Sigma‐Aldrich). The vacuum filling method was used to fill the inside of the microchannel with the BSA solution, due to the limited accessibility of narrow microchannels to liquids [56]. The PDMS chamber was vacuumed and immediately filled by adding BSA solution to the soma compartment, allowing the solution to be aspirated into the channels. After rinsing with sterile water and drying, the chamber was manually aligned and placed onto the MEA surface under an optical microscope. Physical adsorption was used to adhere the PDMS chamber to the MEA. To promote axonal outgrowth, the assembled device was coated with appropriate protein solutions by using vacuum filling. For rDRG‐SNs, a 50 µg/ml laminin solution (Sigma‐Aldrich) was used, whereas for human hPSC‐SNs, SureBond‐XF coating solution (1:200 dilution; Axol Bioscience) was applied. The coating solution was removed immediately prior to cell seeding.

### 4.2 Characterization of GF‐Based Photothermal Heater

The temperature rise of the photothermal heater under laser irradiation was measured using an infrared thermographic camera (R550Pro, Nippon Avionics). To capture the surface temperature of the heater, measurements were conducted in air at room temperature without the PDMS chamber or culture medium. The sampling rate was set at 120 Hz, with a temperature resolution of 0.025 °C. To analyze temporal changes and power‐dependent heating, the average temperature within a 2 × 2 pixel region (40 × 40 µm) surrounding the hottest point was used as the representative value. Optical transmittance of the photothermal heater was evaluated using a spectrophotometer (UH4150, Hitachi High‐Tech Corporation). Raman spectra were obtained using a Raman spectrometer (inVia Qontor, Renishaw) with a 532 nm excitation and a standard grating (1800 lines/mm). Film thickness was measured using atomic force microscopy (AFM; Dimension Icon, Bruker) equipped with a SiN cantilever (Bruker), using a spring constant of 0.4 N/m.

### 4.3 Finite Element Simulation of Temperature Rise

The temperature rise was also evaluated using finite element analysis (COMSOL, COMSOL Multiphysics). The simulation assumed a 1 µm‐thick parylene‐C layer exposed to air, with a graphene interface at the boundary. Surface temperature was estimated under the condition of a 100 µm‐diameter laser spot focused at the center of the graphene layer. The absorption rate of the graphene layer was set to 18%, on the basis of spectrophotometry measurements. Simulations were performed for laser powers of 50, 75, and 100 mW. To improve computational efficiency, radial symmetry around the center of the laser spot was assumed. For millisecond‐scale temperature dynamics, calculations were performed at 0.1 ms intervals, whereas for 1‐second temperature changes, the time step was set to 10 ms. In each simulation, the average surface temperature within the laser spot was computed and used as the representative value.

### 4.4 Cell Culture of Rat DRG Neurons and Human iPSC‐Derived Sensory Neurons

Rat DRG sensory neurons were cultured using a previously reported protocol [42]. DRGs were isolated from embryonic day 15 (E15) Wistar rat embryos (Jackson Laboratory Japan). Pregnant dams were anesthetized with isoflurane and euthanized by cervical dislocation; embryos were rapidly removed and decapitated according to the institutional protocol. Ganglia were dissociated with 0.25% trypsin (Thermo Fisher Scientific) for 15 min at 37°C, followed by gentle trituration to obtain a single‐cell suspension. Cells were seeded into one side of the soma compartment at an initial density of 1000 cells/mm^2^. The culture medium consisted of Neurobasal medium (Thermo Fisher Scientific) supplemented with 0.5 mm glutamine (Sigma‐Aldrich), 25 µm glutamate (Sigma‐Aldrich), 50 µg/mL gentamicin (Thermo Fisher Scientific), 2% B‐27 supplement (Thermo Fisher Scientific), and 100 ng/mL nerve growth factor (NGF; Peprotech). The medium was replaced every 2–3 days by half‐volume exchange with fresh medium. All animal experiments were approved by the Biological Safety and Ethics Committee of NTT Basic Research Laboratories (approval ID: 2025‐05) and were conducted in accordance with the Guidelines for the Proper Conduct of Animal Experiments of the Science Council of Japan (Kohyo‐20‐k16‐2, 2006).

Human iPSC‐derived sensory neuron progenitors (Axol Bioscience) were seeded into one side of the soma compartment at a density of 1000 cells/mm^2^. On the day of seeding, cells were cultured in Neural Plating Medium (Axol Bioscience) supplemented with 10 µm Y‐27632 (Focus Biomolecules), a ROCK inhibitor. After 24 h, the plating medium was completely replaced with fresh maintenance medium. The maintenance medium consisted of Neural Maintenance Medium (Axol Bioscience) supplemented with growth factors [glial cell line‐derived neurotrophic factor (GDNF, 25 ng/mL), nerve growth factor (NGF, 25 ng/mL), brain‐derived neurotrophic factor (BDNF, 10 ng/mL), and neurotrophin‐3 (NT‐3, 10 ng/mL); all from Axol Bioscience], 1% Sensory Neuron Maturation Maximizer Supplement (Axol Bioscience), 1% penicillin–streptomycin (Corning), and 0.25% SureBond‐XF coating solution. On days 3 and 5 post‐seeding, the culture medium was completely refreshed, and thereafter, half‐volume medium changes were performed every 2–3 days. Both rDRG‐SNs and hPSC‐SNs were maintained in a humidified CO_2_ incubator at 37°C with 5% CO_2_.

### 4.5 Image Acquisition

For immunocytochemistry, samples were fixed with 4 wt% paraformaldehyde solution (Sigma‐Aldrich) for 30 min. Cell membrane permeabilization and blocking were then performed by incubating the samples for 3 h in phosphate‐buffered saline (PBS; Thermo Fisher Scientific) containing 1 wt% bovine serum albumin (BSA; Sigma‐Aldrich) and 0.01 wt% Triton X‐100 (Sigma‐Aldrich). Afterward, the solution was replaced with 0.1% BSA solution containing primary antibodies, and the samples were incubated overnight at 4°C. Following three PBS rinses, samples were incubated with secondary antibodies diluted in 0.1% BSA at 4°C for 6–8 h. Nuclear staining was performed by incubating with 10 µg/mL Hoechst 33342 for 30 min at room temperature. The primary antibodies used were anti‐Tau1 (neuronal marker, 1:400 dilution, Novus Biologicals) and anti‐S100β (Schwann cell marker, 1:200 dilution, Abcam). The secondary antibodies used were Alexa Fluor 488 (1:500 dilution, Thermo Fisher Scientific) and Alexa Fluor 546 (1:500 dilution, Thermo Fisher Scientific). Imaging was performed using a confocal laser scanning microscope (SDOSR; Evident Scientific).

Cell viability was assessed using a Live/Dead assay kit (Thermo Fisher Scientific). Live cell nuclei were stained with 10 µg/mL Hoechst 33342 (Thermo Fisher Scientific), live cytoplasm with 2 µm Calcein‐AM, and dead cell nuclei with 4 µm EthD‐1. A continuous‐wave (CW) laser irradiated the samples with an intensity ranging from 38 to 94 mW for 10 sec, followed by staining and incubation for 30 min in a CO_2_ incubator. Samples were rinsed twice with PBS and imaged using the confocal laser scanning microscope (SDOSR; Evident Scientific). Image analysis was performed using the open‐source software ImageJ. All fluorescence images were binarized using Otsu's method [57]. Nuclei were identified using binarized images of Hoechst 33342 and EthD‐1 via the Particle Analyzer plugin in ImageJ. Calcein‐positive area was quantified as the ratio of the binarized Calcein‐AM image area to the total area within the region of interest (ROI), which was defined as the 100 × 100 µm photothermal heater region.

Samples for SEM were fixed with 2 wt% glutaraldehyde solution (Fujifilm Wako Chemicals). After replacing PBS with distilled water, the solvent was gradually exchanged with ethanol, and then with tert‐butyl alcohol (Fujifilm Wako Chemicals). Samples were placed at −20°C for 30 min, followed by sublimation of the tert‐butyl alcohol under vacuum using a freeze dryer (FS‐2030, EYELA). Finally, a gold sputter coating was applied using an ion sputter gun (E‐1030, Hitachi), and samples were imaged using a focused ion beam–scanning electron microscope (FIB‐SEM; Auriga 60 Cross Beam Workstation, Carl Zeiss).

### 4.6 Extracellular Recording and Photothermal Stimulation Setup

The experimental setup for simultaneous extracellular recording and photothermal stimulation was based on the MEA control system (MED64‐Basic System, Alpha Med Scientific) (Figure S1). The MEA control system consists of a main amplifier, a head amplifier, and a connector, enabling simultaneous recording of extracellular potentials and current pulse stimulation. Electrical pulses were delivered to a laser driver (CLD1015, Thorlabs), which powered a near‐infrared laser diode (785 nm; FPL785P, Thorlabs). This configuration allowed the laser output to be synchronized with the amplitude and duration of the current pulses. The laser beam was focused onto the MEA surface using a collimator (CFP2‐780A, Thorlabs) and a plano‐convex lens. The laser was directed onto the MEA substrate at a 45° angle from below through the stage‐top incubator (Tokai Hit), which maintained the cellular environment. The irradiation position was manually controlled using a three‐axis micromanipulator holding the collimator and lens assembly. The recorded signals were amplified 2000‐fold, band‐pass filtered between 100 and 2000 Hz, and digitized at a sampling rate of 20 kHz. Throughout the experiments, the culture environment was maintained at 37°C and 5% CO_2_ by using the stage‐top incubator.

### 4.7 Experimental Protocol for Recording Axonal Thermal Responses

Axonal responses to thermal stimulation were measured by running programs in the MEA control system. To stabilize the sample temperature, the NoC device was transferred from the CO_2_ incubator to the stage‐top incubator and left to equilibrate for 10 min before recording began. Under a phase‐contrast microscope, the laser beam (<30 mW) was manually positioned over the photothermal heater, and the stimulation program was then initiated. In the CW laser stimulation experiments with constant intensity, spontaneous activity was recorded for 10 sec, followed by 10 sec of CW laser irradiation. For experiments with gradually increasing laser intensity, the power was incremented every 2 sec. In the pulsed laser stimulation experiments at constant intensity, spontaneous activity was recorded for 10 sec, followed by 100 cycles of 1 ms‐wide laser pulses delivered at 10 Hz. For the stimulation protocols with increasing intensity, laser power was raised incrementally at 1 Hz, with one power step per pulse. After each stimulation trial, the laser spot was moved to the next microchannel, and the procedure was repeated. Eight microchannels were measured per MEA, and more than five MEAs were tested for each experimental condition.

### 4.8 Signal Processing

Signal processing was performed using custom codes in MATLAB (MathWorks). Spikes indicating APs were detected by identifying negative peaks that exceeded five times the standard deviation of the baseline signal. Electrodes showing at least a twofold increase in firing rate after stimulation compared with the baseline were classified as active and included in subsequent analyses. Results of signal processing were aggregated for each microchannel. For firing rate calculations, the mean firing frequency across the eight electrodes within each microchannel was used as the representative value. In continuous stimulation experiments, rise time and decay time of firing were defined as the time intervals from the onset to 10% and from the peak to 20% of the peak firing rate, respectively. For repeated pulsed stimulation, response probability was calculated by dividing the number of microchannels that showed a response to each individual pulse by the total number of responsive channels that responded at least once across 100 stimuli. Action potential propagation was assessed based on the time difference between responses recorded on electrode no. 3 and electrode no. 6. Events with response time differences within a 10‐ms window were considered to represent AP propagation. Conduction velocity was calculated by dividing the propagation distance (1350 µm) by the response time difference. Additionally, the AP initiation time at the stimulation site was estimated using both the distance between the heater and electrode no. 6 (675 µm) and the conduction velocity (Figure S14).

### 4.9 Statistical Analysis

Due to variability in neural responses across microchannels, all representative values and plotted data for these measurements are presented as mean ± standard error (SE). For all other analyses, results are expressed as mean ± standard deviation (SD). Statistical significance between groups was determined using Welch's *t*‐test, with a significance threshold set at *p* < 0.05.

## Funding

This work was supported by Grants‐in‐Aid for Scientific Research (JP25H02631) from the Japan Society for the Promotion of Science.

## Conflicts of Interest

The authors declare no conflict of interest.

## Supporting information




**Supporting File**: smll73577‐sup‐0001‐SuppMat.pdf.

## Data Availability

The data that support the findings of this study are available from the corresponding author upon reasonable request.
