# Supplementary material for: In Vitro Reconstruction of Axonal Heat Sensing with a Photothermal Nerve‐on‐a‐Chip
Source: Small. 2026 Apr 30;22(45):e73577. doi: 10.1002/smll.73577 (PMC13472297; doi:10.1002/smll.73577)
Supplement: Supplementary file 1 [file SMLL-22-e73577-s001.pdf]

Supporting Information

In Vitro Reconstruction of Axonal Heat Sensing with a Photothermal Nerve-on-a-Chip

Koji Sakai,[1,2] Yujiro Tanaka,[2,3] Riku Takahashi,[1,2] Toichiro Goto,[1,2] Yosuke Mizuno,[1,2] Taiki Otomo,[1,2] and Aya Tanaka[1,2]*

This file includes

- Supplementary Figures S1 to S17
- Supplementary Table S1 to S4

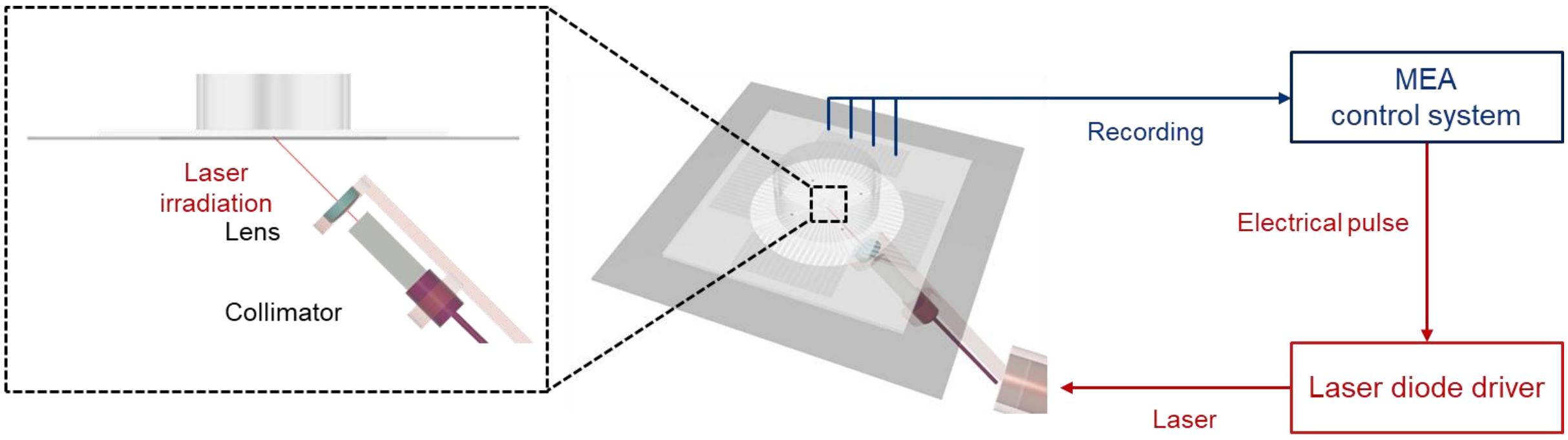

Figure S1 | Experimental setup for extracellular recordings and photothermal stimulation. To synchronize extracellular recordings with photothermal stimulation, both were controlled by the MEA control system. Electrical pulses were delivered to a laser diode driver, which controlled the timing and amplitude of the laser output. The laser beam was focused using a collimator and a lens, with the distance between them adjusted using a 3D-printed fixture. The position of the fixture was precisely adjusted using a three-axis micromanipulator.

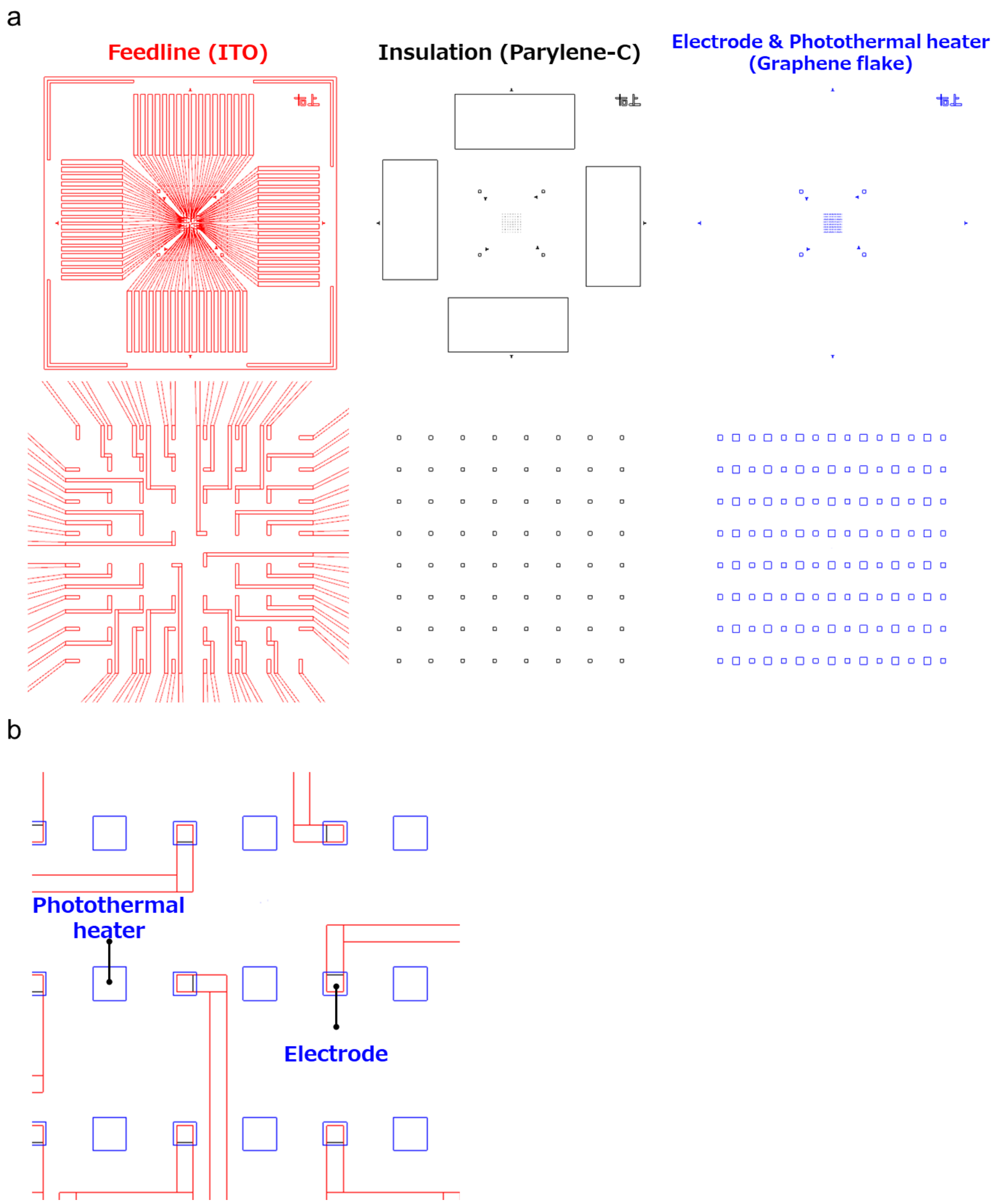

Figure S2 | Design of the MEA substrate. a) CAD patterns of the individual layers. The upper panel shows the overall layout, and the lower panel shows a magnified view of the electrode area. **b)** Merged CAD image of the electrodes and photothermal heaters. Each electrode was positioned at the end of the ITO feedline, and the photothermal heaters were aligned between the electrodes.

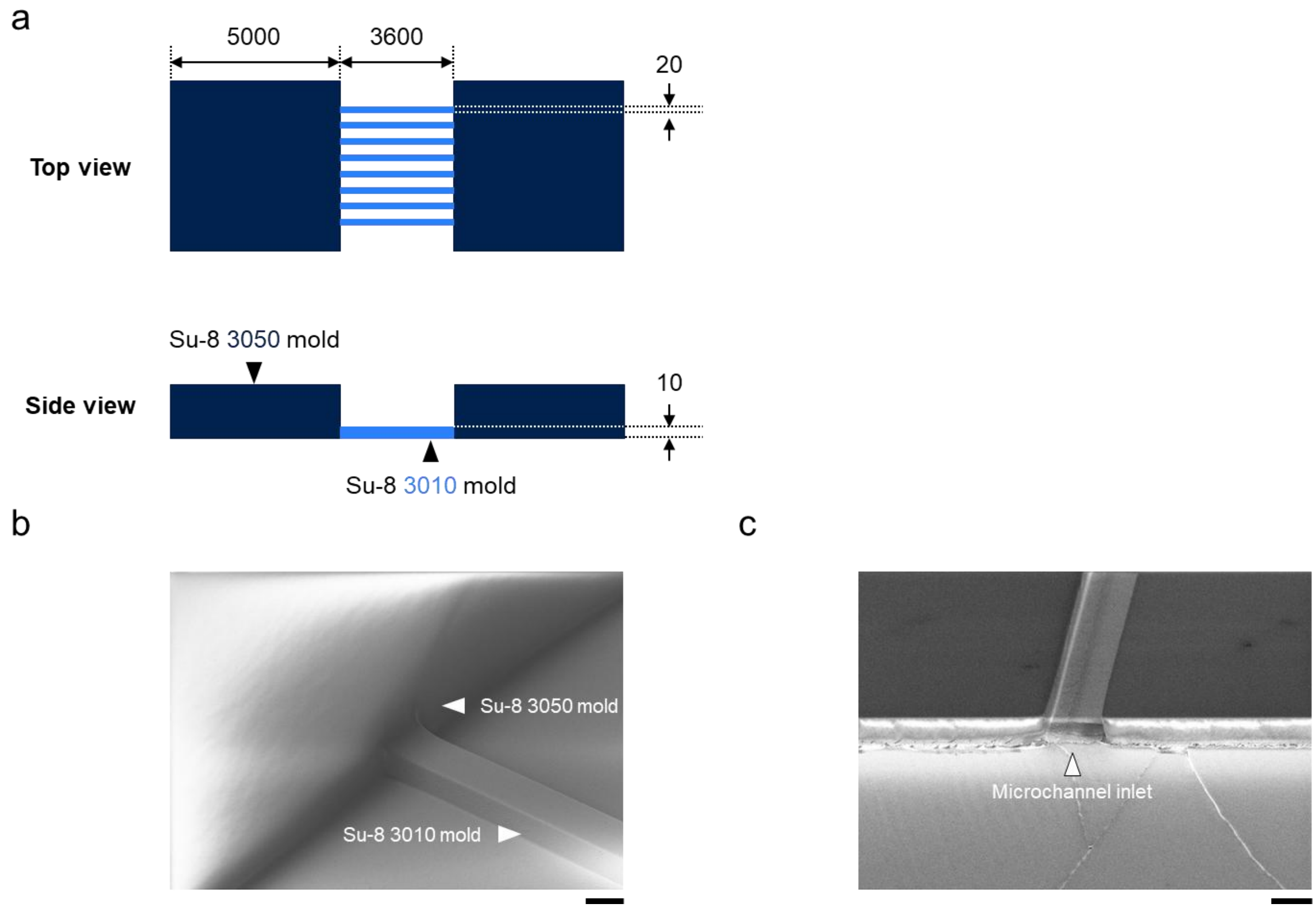

Figure S3 | Design of SU-8 mold for PDMS chamber. a) Schematic of the SU-8 mold, which has two steps to isolate axons from neuronal somata. The lower structure was fabricated using SU-8 3010, and the higher structure was fabricated using SU-8 3050. **b)** SEM image of the SU-8 mold at the inlet of the microchannel. **c)** SEM image of the PDMS chamber at the inlet of the microchannel. Scale bars, 10 µm.

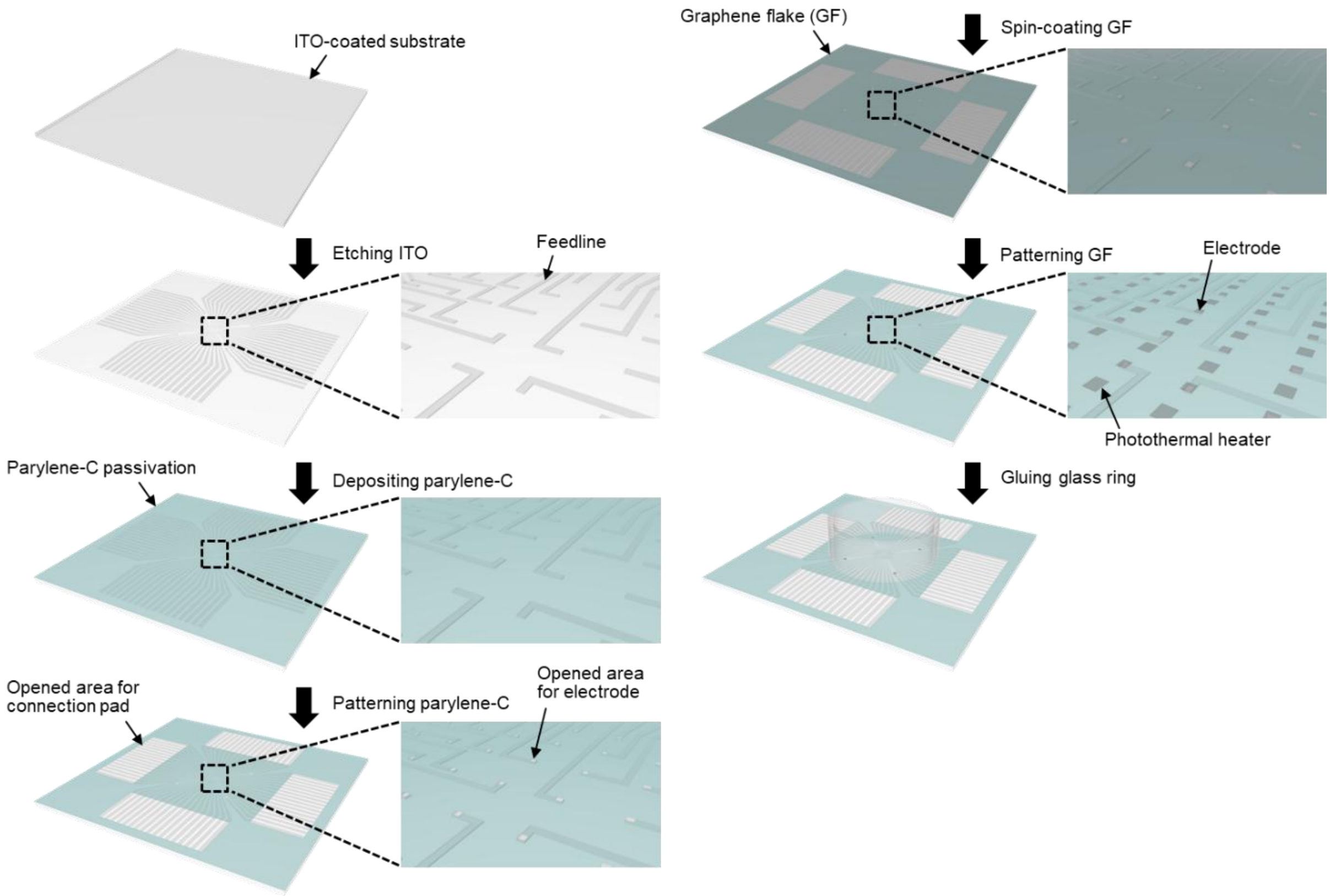

Figure S4 | Fabrication process of MEA substrate. First, the ITO-coated substrate was etched with an ITO etchant through a photoresist pattern to form the feedlines. Second, the ITO feedlines were covered with a 1.5-µm-thick parylene-C layer. Third, the areas corresponding to the connection pads and electrodes were opened by RIE with O_2 plasma. Next, graphene flakes (GFs) were spin-coated and patterned by brief RIE. Finally, a glass ring for retaining the culture medium was placed on the surface.

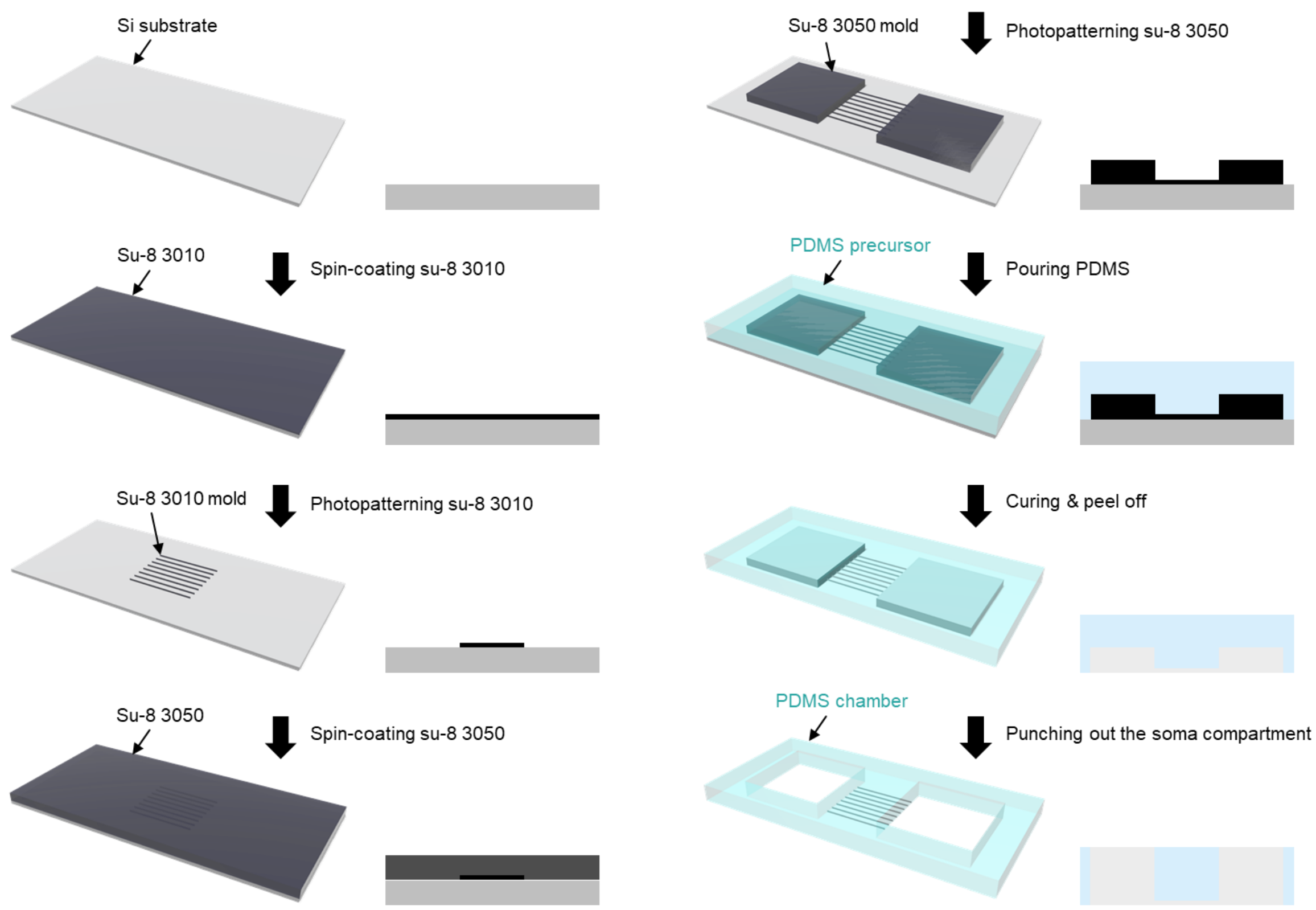

Figure S5 | Fabrication process of PDMS chamber. First, SU-8 3010 was spin-coated onto a Si substrate and patterned by UV exposure to form the microchannel mold. Next, SU-8 3050 was spin-coated and patterned to form the soma compartment mold. To facilitate detachment of the PDMS chamber, the mold was coated with a 1-µm-thick parylene-C layer. After pouring the PDMS precursor onto the mold, the PDMS was cured, detached, and then punched to create the soma compartments.

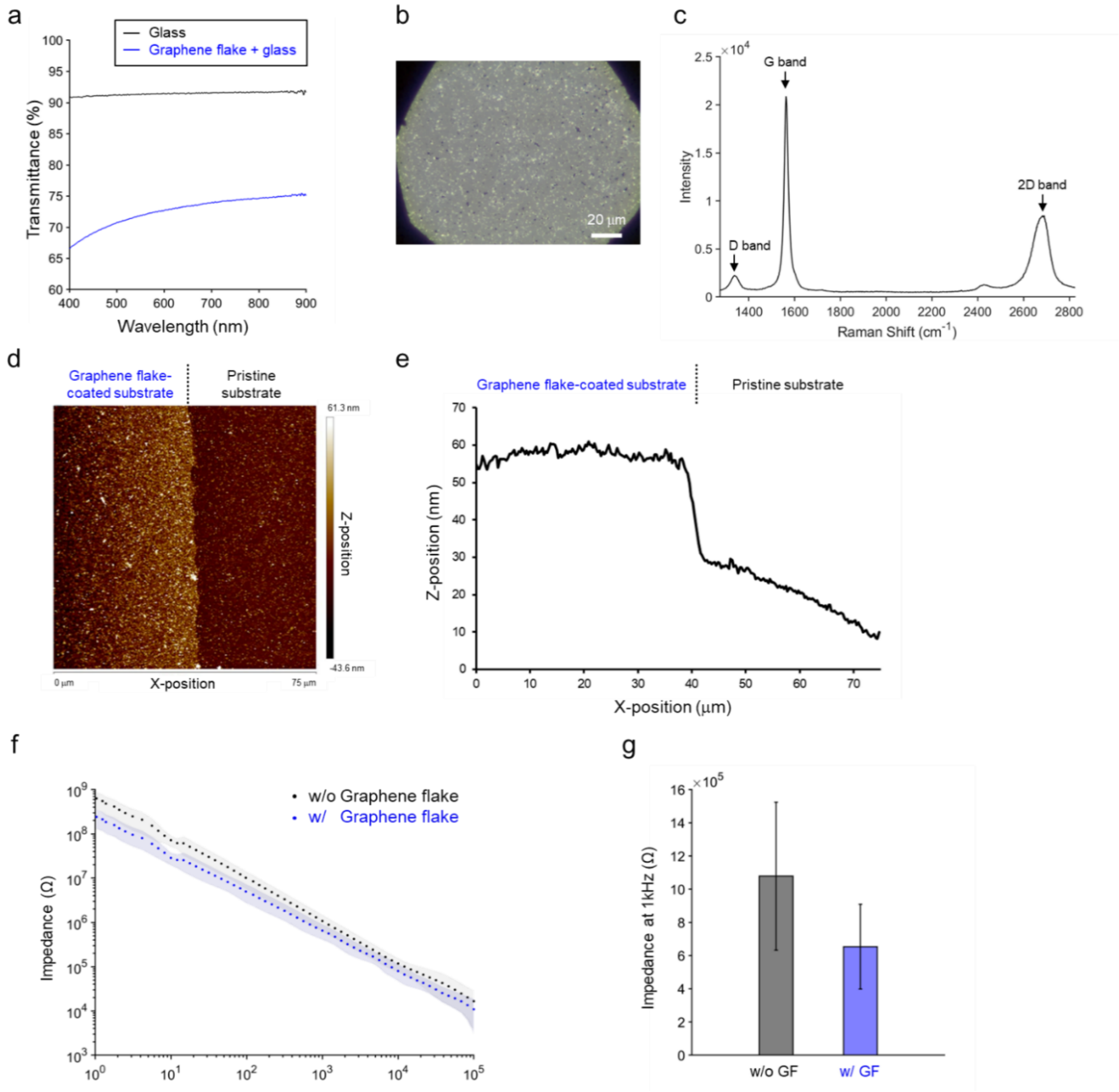

Figure S6 | Material properties of graphene flakes. a) Transmittance of graphene-flake (GF)-coated and pristine glass, showing that GFs absorb 18% of light at 785 nm. **b)** Optical microscopic image of GF-coated glass under the Raman spectroscopy system. **c)** Raman spectrum of GFs, showing prominent graphene peaks at the G and 2D bands with a moderate peak at the D band. **d)** AFM topography image at the boundary between GF-coated and pristine surfaces. **e)** Height profile at the boundary between GF-coated and pristine surfaces. **f)** Electrode impedance of ITO electrodes with (w/) and without (w/o) GF, showing a slight decrease after GF coating. **g)** Comparison of impedance at 1 kHz between w/ and w/o GF. Data are presented as mean ± SD (n = 7 electrodes).

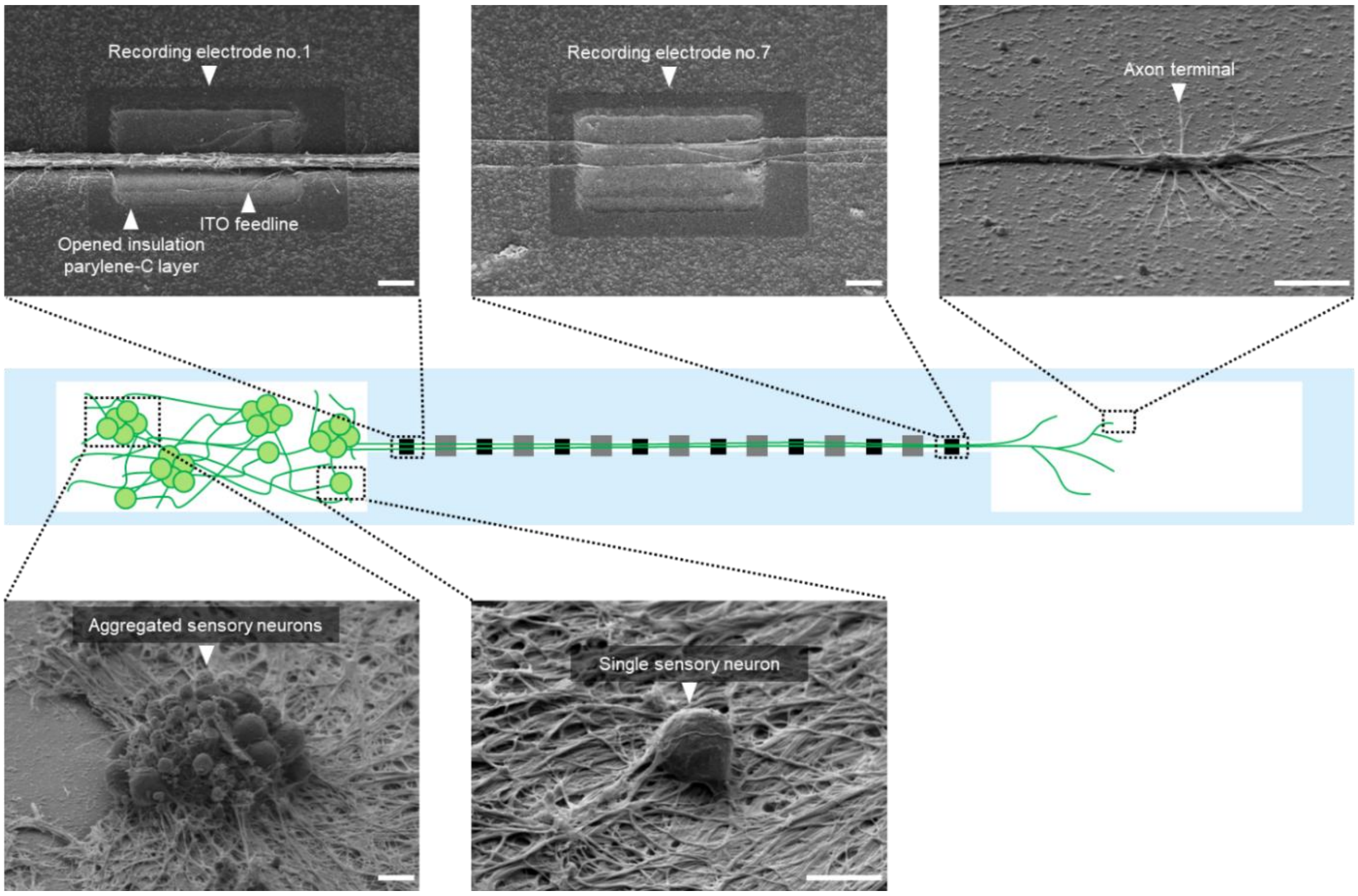

Figure S7 | SEM images of sensory neurons grown in the NoC device. SEM images were obtained from hPSC-SNs cultured in a NoC device. As shown in the lower images, hPSC-SNs tended to aggregate while extending their axons randomly within the soma compartment. In the upper images, axons can be seen growing through the microchannel, crossing over the electrodes and photothermal heaters. The number of axons in the bundle decreased with increasing distance from the soma compartment. In another compartment, axon terminals were observed. Scale bars, 10 µm.

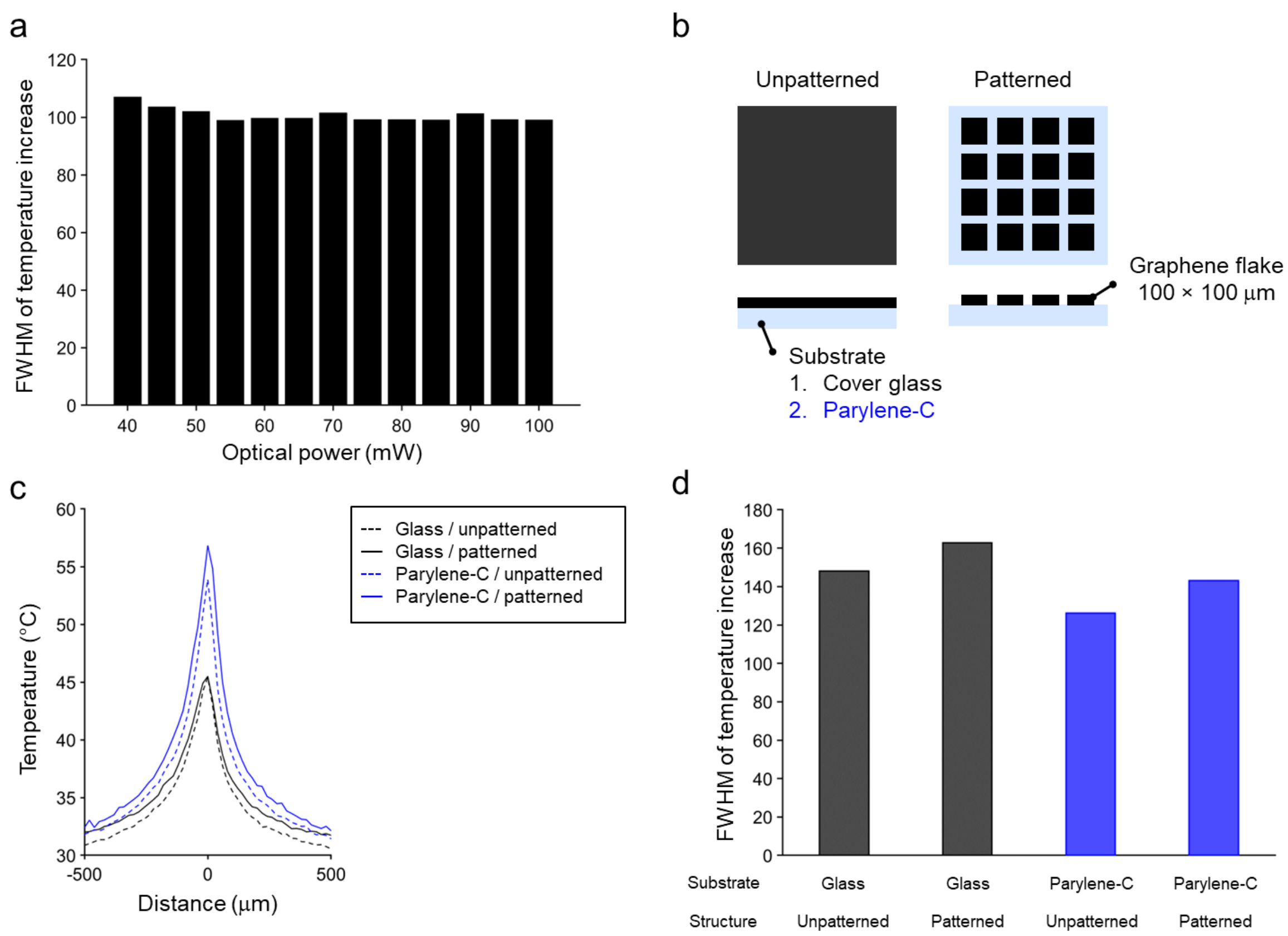

Figure S8 | Supplementary temperature characterization of GF-based photothermal heater. a) FWHM of the lateral temperature profile at varying optical powers, showing a constant value of 100 μm regardless of laser power. **b)** Schematics of unpatterned and patterned GFs used to compare heating spot size. The underlying substrate was either parylene-C-coated or pristine cover glass. **c)** Lateral temperature profiles of four types of photothermal heater, indicating more efficient heating on the insulating parylene-C. **d)** FWHM comparison showing that the underlying insulator contributes to localized heating, whereas GF patterning does not sharpen the heating profile.

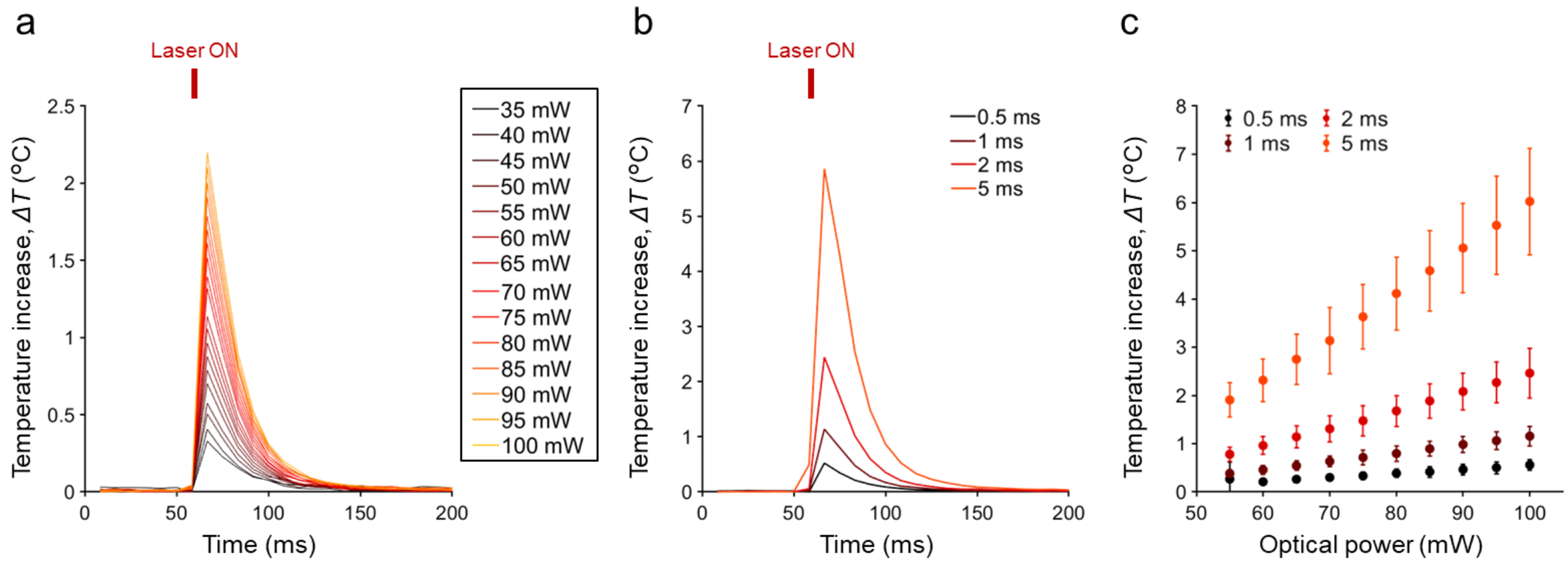

Figure S9 | Temperature characterization upon millisecond-duration laser irradiation using a thermographic camera. a) Temperature increment after 1-ms pulse laser irradiation at varying laser powers, showing a sharp decrease with a half-life of 10–20 ms. **b)** Temperature increment after several-millisecond pulse laser irradiation at 100 mW. **c)** Peak temperature increase at different laser powers and pulse widths, indicating that longer and stronger pulses generate higher temperature increments. Note that, since the sampling rate of the thermographic camera is 120 Hz, the measured peak temperature is slightly lower than the actual value. Data are presented as mean ± SD (*n* = 96 trials).

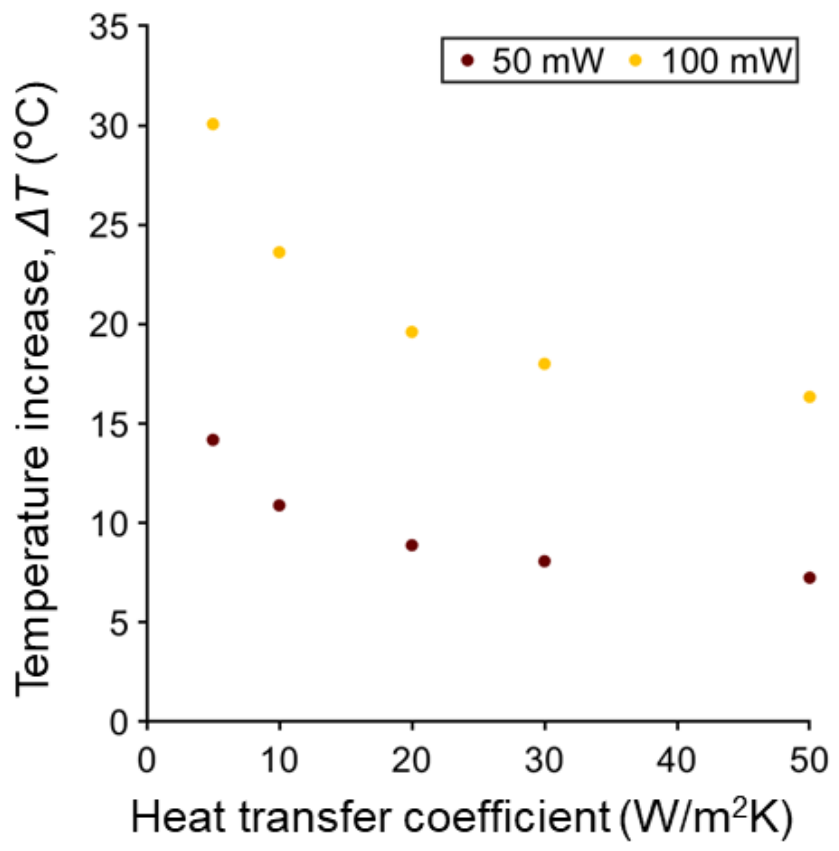

Figure S10 | Effect of heat transfer coefficient on temperature rise in finite-element simulations. The temperature increase at 1000 ms during continuous laser heating is plotted as a function of the heat transfer coefficient (h = 5–50 W/m^{2}K) for two laser powers (50 and 100 mW). The heat transfer coefficient represents the effective heat dissipation to the surrounding environment. Values of h ≈ 50 W/m^{2}K correspond to conditions where the device is surrounded by water, while intermediate values (h ≈ 20–30 W/m^{2}K) approximate environments involving both aqueous medium and PDMS. In contrast, h = 5 W/m^{2}K represents air. The simulations show that increased heat dissipation reduces the temperature rise. Under aqueous conditions, the temperature increase is estimated to be approximately 60-70% of that obtained in air. These results provide a conservative estimate of the thermal conditions under cell culture environments.

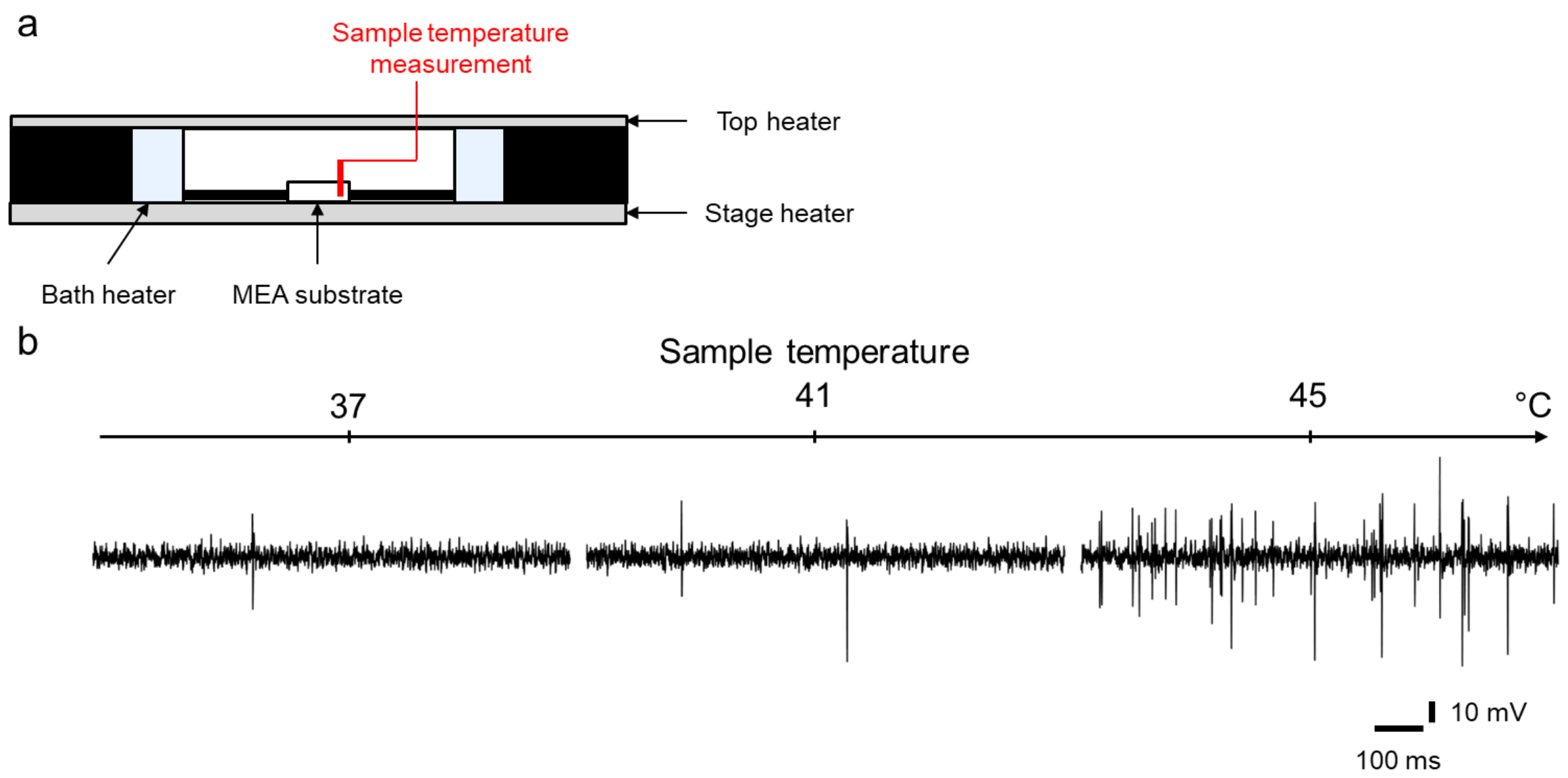

Figure S11 | Thermal stimulation to rDRG SNs using a stage-top incubator system. a) Experimental setup of MEA recording under controlled bath temperature. The sample temperature was measured using a thermometer, and the three heaters equipped in the stage-top incubator were adjusted accordingly. **b)** Representative traces of extracellular potentials with increasing sample temperature. The firing frequency markedly increased when the temperature exceeded 42 °C, the activation threshold of the TRPV1 channel, confirming the responsiveness of rDRG SNs to heating above the TRPV1 threshold.

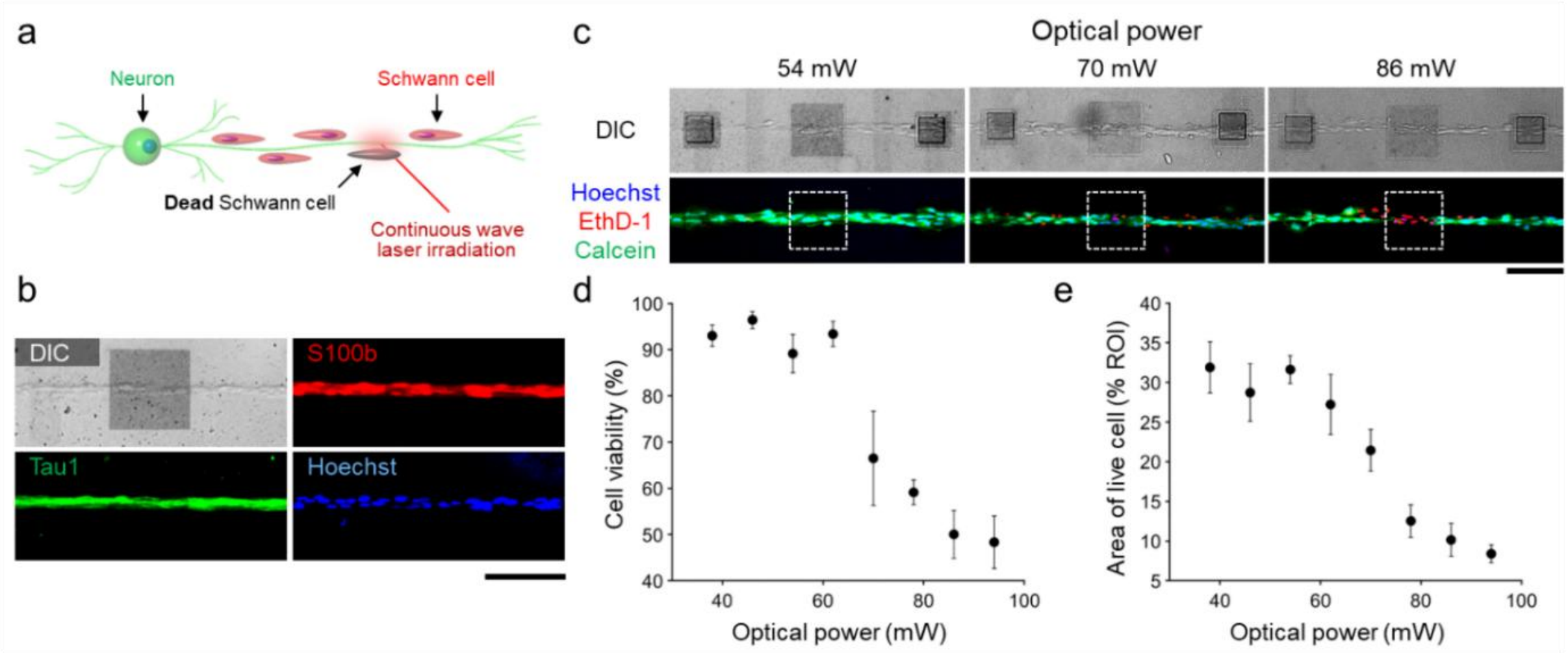

Figure S12 | Evaluation of safe photothermal stimulation conditions using cell viability assays. **a)** Protocol for evaluating cell viability in rat DRG neuron cultures after 2 s laser stimulation. **b)** Immunocytochemical and differential interference microscope (DIC) images of Schwann cells (S100b; red) in microchannels Because Schwann cells in DRG culture are smaller than neurons, they can migrate into the micro-channels, allowing for viability assay within the channels. **c)** Fluorescent live/dead staining under different laser intensities. Live cells were identified by Hoechst 33342 (blue) and Calcein-AM (green) staining, whereas dead cells were labelled with Ethidium Homodimer-1 (EthD-1; red). The white dotted boxes indicate the region of interest (ROI) according to the heater. **d, e)** Quantification of cell viability and Calcein-positive area in ROI, showing that cell damage increases above 70 mW. Note that continuous stimulation below 60 mW preserves cell viability (>85%). Data are shown as mean ± SD (*n* = 8 heaters). Scale bars, 100 µm.

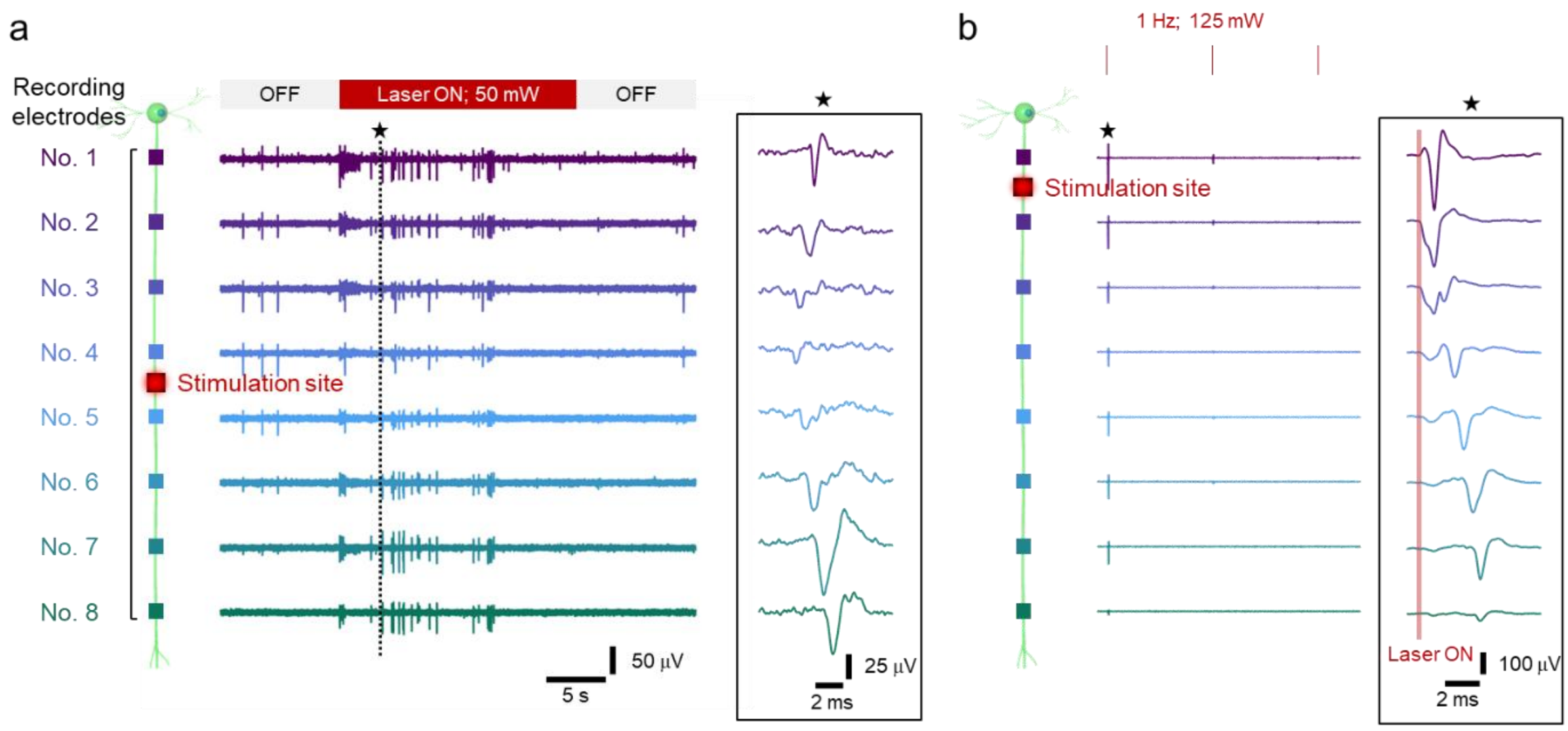

Figure S13 | Action potential propagation from different stimulation sites. a) Representative traces during continuous photothermal stimulation of the heater between electrodes no. 4 and 5. As shown in the enlarged view (★), evoked action potentials propagated bidirectionally from the stimulation site. **b)** Representative traces during 1-ms pulse stimulation of the heater nearest to the soma. The pulse-locked response propagated orthodromically along the axons from the stimulation site (★) and decreased rapidly, likely due to TRPV1 desensitization.

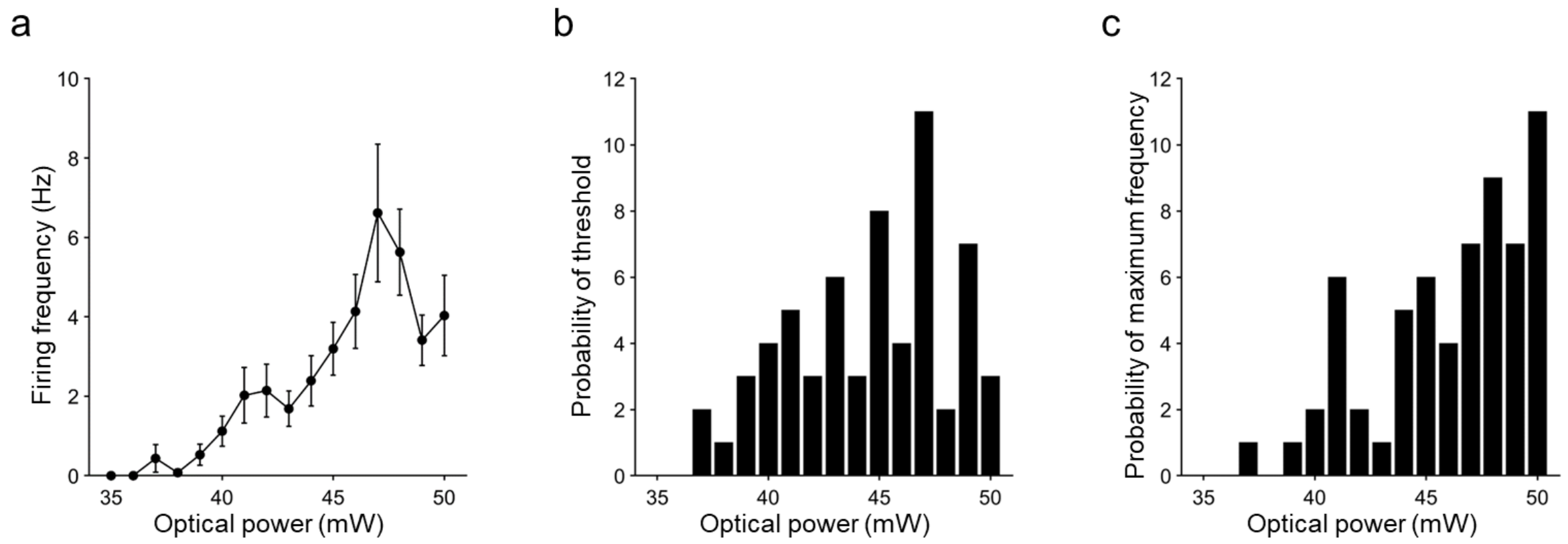

Figure S14 | Supplementary information on responsiveness to continuous laser stimulation. a) Averaged changes in firing frequency with gradually increasing laser power. **b)** Histogram of the threshold laser power at which APs were evoked. **c)** Histogram of the laser power at which the firing frequency reached its maximum. Data are presented as mean ± SE (*n* = 62 microchannels).

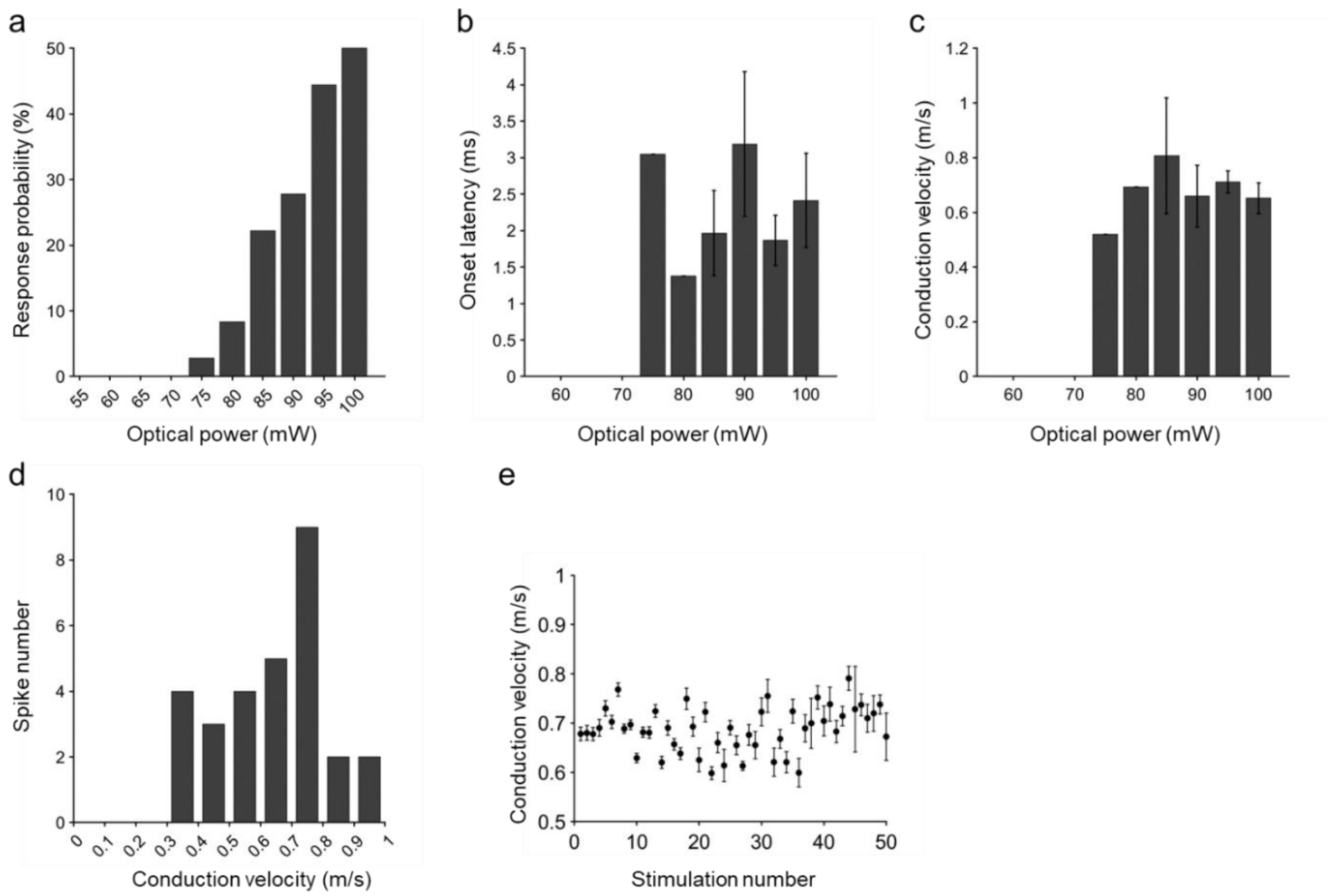

Figure S15 | Supplementary information on responsiveness to 1-ms pulse stimulations. a) Response probability of pulse-locked responses to 1-ms pulse stimulation at 1 Hz with increasing laser power. **b)** Onset latency of pulse-locked responses at different laser powers. **c)** Conduction velocity of pulse-locked responses versus laser power. **d)** Histogram of conduction velocity, peaking at 0.7–0.8 m/s, which is a typical value for rDRG SNs. **e)** Conduction velocity remained essentially unchanged during repeated 1-ms pulse stimulations. Data are presented as mean ± SE (n = 32 microchannels for **a–d**; n > 3 microchannels for each stimulation number in **e**).

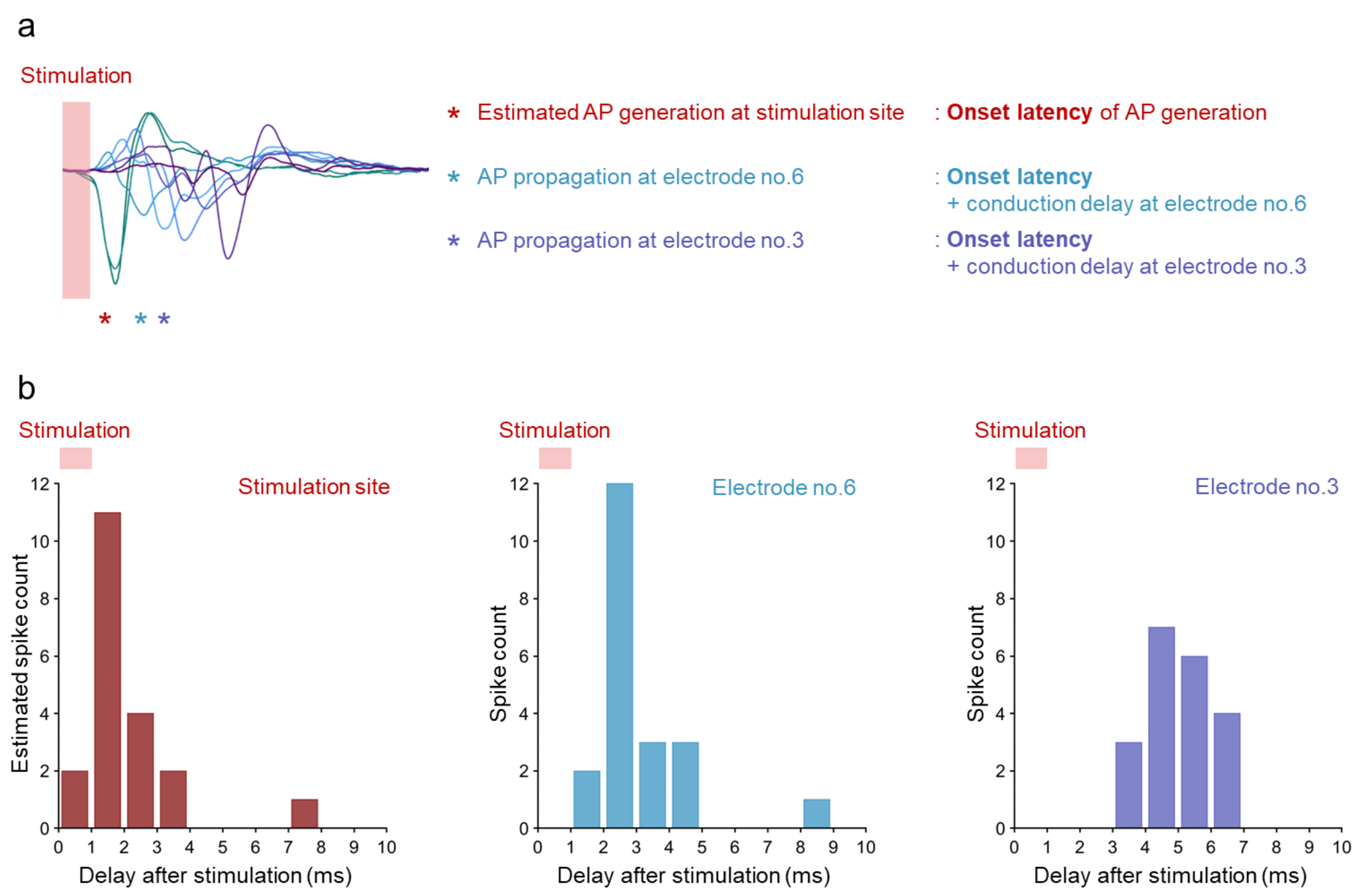

Figure S16 | Estimation of onset latency of AP initiation at the stimulation site. a) The delay of the pulse-locked response includes both the onset delay of AP generation and the conduction delay between the stimulation site and the recording electrode. Therefore, the onset delay can be calculated by back-extrapolating from the spike timings at two electrodes. **b)** Histogram of the estimated onset delay at the stimulation site, along with the measured response timings at electrodes no. 3 and 6.

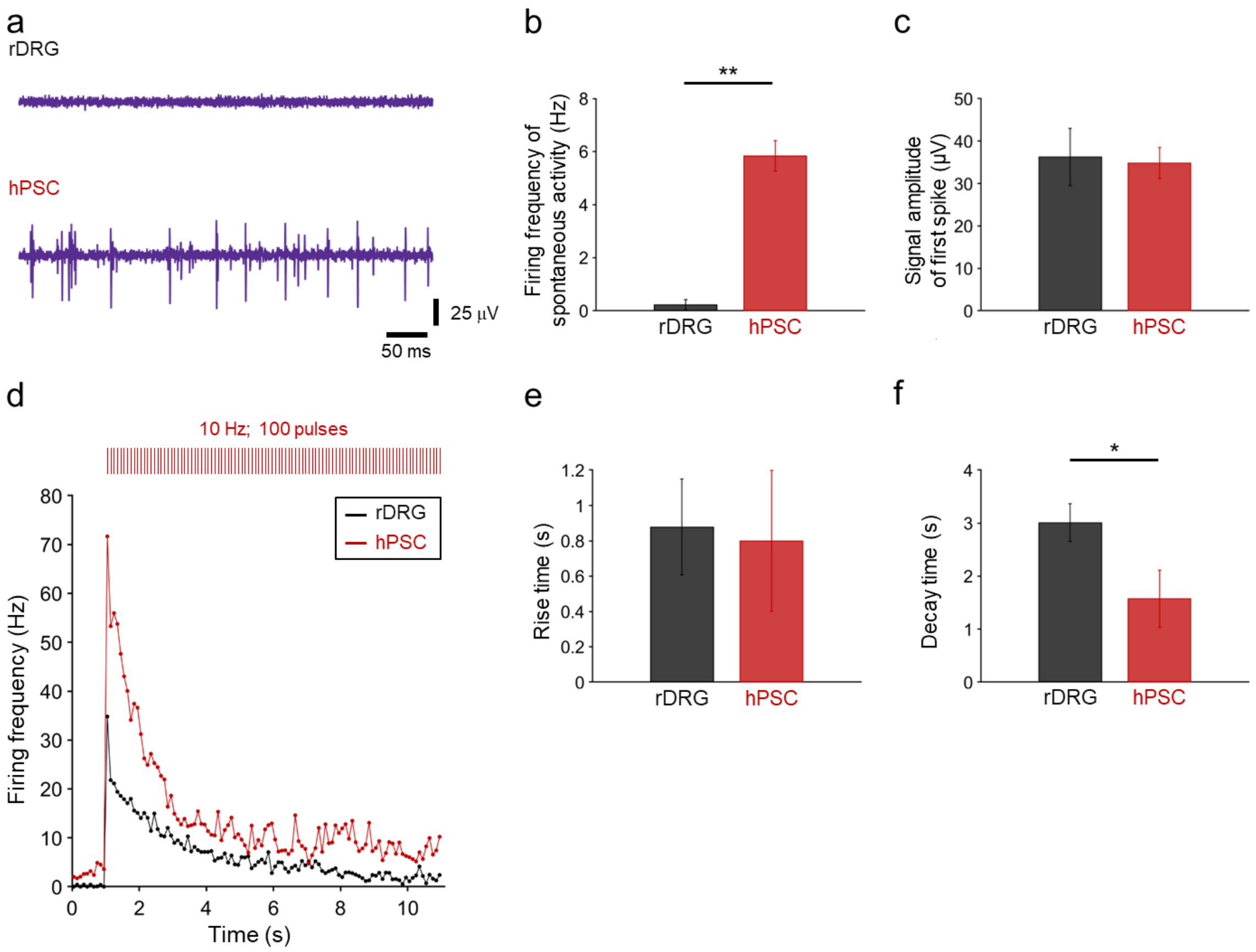

Figure S17 | Comparison of thermal responsiveness between rDRG SNs and hPSC SNs. **a)** Representative traces of rDRG and hPSC SNs before stimulation, showing prominent spontaneous activity in hPSC SNs. **b)** Significantly higher spontaneous firing frequency in hPSC SNs. **c)** Comparable first-spike amplitudes between rDRG-SNs and hPSC-SNs during repeated 1-ms photothermal pulses. **d)** Rapid decrease in firing frequency of hPSC SNs during repeated 1-ms pulse stimulations, indicating that inter-pulse firing decreased with thermoreceptor desensitization. **e, f)** Rise time and decay time of firing frequency upon repeated 1-ms pulse stimulation at 10 Hz. Data are presented as mean ± SE. n = 22 (rDRG) and 38 (hPSC) microchannels for **b**; n = 18 (rDRG) and 11 (hPSC) microchannels for **c–f**. *$p < 0.05$ and **$p < 0.01$; Welch's t-test.

Table S1 Design of Microtunnels.

Length (μm)	Width (μm)	Height (μm)	Interval (μm)
3600	20	10	450

Table S2 Design of MEA substrate.

Width of feedline (μm)	GF-coated area for electrode (μm^2)	Electrode interval (μm)	Heating element (μm)	Open area of parylene-C (μm^2)	Reference electrode (μm^2)
50	70×70	450	100×100	50×50	500×500

Table S3 Measurements of PDMS microchannel and su-8 mold.

	Width (μm)	Thickness (μm)
Su-8 3010 mold for microchannel	18.21 ± 0.23	9.90 ± 0.52
Su-8 3050 mold for soma compartment		104.76 ± 10.42
PDMS microchannel	17.17 ± 0.72	8.20 ± 0.44

Table S4 Optical absorbance of individual materials in MEA.

	Glass substrate	ITO	ParyleneC	Single layer graphene	Graphene flake
%T	92.0	92.8	99.1	99.6	82.3
%Abs	8.0	7.3	0.9	0.4	**17.5**
